# Plasma pTau181 and amyloid markers predict conversion to dementia in idiopathic REM sleep behaviour disorder

**DOI:** 10.1093/brain/awaf003

**Published:** 2025-01-06

**Authors:** Aline Delva, Amélie Pelletier, Emma Somerville, Jacques Montplaisir, Jean-François Gagnon, Gwendlyn Kollmorgen, Tony Kam-Thong, Thomas Kustermann, Venissa Machado, Ziv Gan-Or, Ronald B Postuma

**Affiliations:** The Neuro (Montreal Neurological Institute-Hospital), McGill University, Montreal, QC H3A 2B4, Canada; Department of Neurology and Neurosurgery, McGill University, Montreal, QC H3A 2B4, Canada; Centre for Advanced Research in Sleep Medicine, Hôpital du Sacré-Coeur de Montréal, Montreal, QC H4J 1C5, Canada; Department of Neurology, Research Institute of McGill University Health Centre, Montreal, QC H4A 3J1, Canada; The Neuro (Montreal Neurological Institute-Hospital), McGill University, Montreal, QC H3A 2B4, Canada; Department of Human Genetics, McGill University, Montreal, QC H3A 2B4, Canada; Centre for Advanced Research in Sleep Medicine, Hôpital du Sacré-Coeur de Montréal, Montreal, QC H4J 1C5, Canada; Centre for Advanced Research in Sleep Medicine, Hôpital du Sacré-Coeur de Montréal, Montreal, QC H4J 1C5, Canada; Department of Psychology, Université du Québec à Montréal, Montreal, QC H2L 2C4, Canada; Roche Diagnostics GmbH, Penzberg 82377, Germany; Roche Pharma Research and Early Development, Pharmaceutical Sciences, Roche Innovation Center Basel, F. Hoffmann-La Roche Ltd, Basel 4052, Switzerland; Roche Pharma Research and Early Development, Neurosciences and Rare Diseases, Roche Innovation Center Basel, F. Hoffmann-La Roche Ltd, Basel 4052, Switzerland; Roche Pharma Research and Early Development, Neurosciences and Rare Diseases, Roche Innovation Center Basel, F. Hoffmann-La Roche Ltd, Basel 4052, Switzerland; The Neuro (Montreal Neurological Institute-Hospital), McGill University, Montreal, QC H3A 2B4, Canada; Department of Neurology and Neurosurgery, McGill University, Montreal, QC H3A 2B4, Canada; Department of Human Genetics, McGill University, Montreal, QC H3A 2B4, Canada; The Neuro (Montreal Neurological Institute-Hospital), McGill University, Montreal, QC H3A 2B4, Canada; Department of Neurology and Neurosurgery, McGill University, Montreal, QC H3A 2B4, Canada; Centre for Advanced Research in Sleep Medicine, Hôpital du Sacré-Coeur de Montréal, Montreal, QC H4J 1C5, Canada; Department of Neurology, Research Institute of McGill University Health Centre, Montreal, QC H4A 3J1, Canada

**Keywords:** dementia with Lewy bodies, blood markers, pTau181, Aβ42, idiopathic/isolated REM sleep behaviour disorder

## Abstract

Blood-based biomarkers for Alzheimer’s disease (AD) pathology have been investigated intensively as markers for AD-related neurodegeneration. Comorbid AD pathology is common in dementia with Lewy bodies (DLB). Accordingly, we hypothesized that plasma biomarkers associated with AD pathology might be useful to predict DLB in a cohort of idiopathic/isolated REM sleep behaviour disorder (iRBD), an incipient synucleinopathy. The aim of this study was to determine whether plasma amyloid-β and pTau181 biomarkers can predict DLB.

This longitudinal single-centre (Canada) cohort study included 158 individuals with polysomnography-confirmed iRBD between September 2004 and October 2022, each providing blood plasma samples, who were then offered prospective follow-up. Plasma Aβ40, Aβ42 and pTau181 levels were measured using NeuroToolKit, a prototype assay panel of neurodegeneration (Roche Diagnostics International Ltd). The primary outcome was the association between plasma biomarkers at baseline and eventual development of DLB. Correlations between plasma markers and baseline cognitive tests were assessed.

A total of 142 iRBD participants [109 male (77%); mean ± SD, 67.6 ± 8.0 years of age] were included in the final analysis. On prospective follow-up (2.9 ± 2.1 years after sampling), 32 individuals phenoconverted to a defined neurodegenerative syndrome (18 DLB, 13 Parkinson’s disease and one multiple system atrophy). The combined phenoconvertor group had lower baseline plasma Aβ42/40 ratio compared with non-phenoconvertors (mean ± SD, 0.103 ± 0.010 versus 0.114 ± 0.012, *P* < 0.001) and higher pTau181 levels (0.993 ± 0.354 versus 0.784 ± 0.266 pg/ml, *P* = 0.008). When divided by phenoconversion subtype, significant differences were seen selectively in DLB convertors [Aβ42/40 = 0.101 ± 0.010, difference −0.011, 95% confidence interval (−0.016; −0.005), *P* < 0.001; pTau181 = 1.144 ± 0.326 pg/ml, difference 0.282 pg/ml, 95% confidence interval (0.146; 0.418), *P* < 0.001]. Cross-sectional analysis showed that plasma pTau181 (but not Aβ42/40) was correlated with cognitive tests across various domains.

Our results indicate that plasma Aβ42/40 ratio and pTau181 can predict conversion to DLB in iRBD.

## Teaser

Delva *et al.* show that blood-based tests for Alzheimer’s disease biomarkers—specifically the plasma Aβ42/40 ratio and pTau181—can be used to predict conversion to dementia with Lewy bodies in individuals with idiopathic/isolated REM sleep behaviour disorder.

## Introduction

With recent development of amyloid-targeting therapies, it has become crucial to detect Alzheimer’s disease (AD) pathology early, reliably and non-invasively. Biomarkers can add crucial supplemental information to clinical examinations, because clinical examinations can be discordant with underlying pathology.^1-3^ Although CSF and PET tracers are established identifiers of AD, their cost and/or invasiveness limits their use. In contrast, blood-based tests would be a major advance, because they could berapidly scalable. Plasma biomarkers such as higher phosphorylated tau (pTau) and lower Aβ42/40 ratios can differentiate AD from both cognitively unimpaired individuals and those with non-AD neurodegenerative disorders.^4-8^ Both plasma pTau181 and Aβ42/40 are correlated with clinical disease severity in AD and with cognitive decline in cognitively normal elderly.^9,10^ Moreover, both pTau181 or pTau217 and combinations of plasma Aβ42/40 and pTau217 markers miight predict progression to Alzheimer’s dementia in cognitively unimpaired individuals.^11,12^

Dementia with Lewy bodies (DLB) is a condition characterized by deposition of α-synuclein pathology in Lewy bodies, often in association with amyloid-β pathology.^13,14^ Studies on CSF of DLB individuals found an abnormal profile of AD-related biomarkers.^15,16^ However, data on blood-based AD-related biomarkers in DLB are ambiguous. One study could not differentiate DLB from controls based on plasma pTau181 and Aβ42/40,^17^ whereas two other studies demonstrated higher plasma pTau181 in DLB and mild cognitive impairment with Lewy bodies (MCI-LB).^18-20^ Another recent study showed that in DLB plasma pTau181 levels were higher compared with healthy controls, but lower than in AD, whereas plasma Aβ42/40 only differentiated DLB from AD^21,22^ (note that plasma pTau181 might be less specific for AD pathology, because recent studies have found elevation of plasma pTau181 in amyotrophic lateral sclerosis and spinal muscular atrophy).^23,24^ Plasma Tau181 and/or Aβ42/40 adequately differentiated between DLB CSF Aβ+ and DLB CSF Aβ−.^21,22^ Moreover, within DLB, higher plasma pTau181 and pTau217 levels were associated with worse cognition.^18,21,22^ Most critically, however, it remains unclear whether blood-based pTau and/or amyloid-β markers can identify early stages of DLB, before dementia onset.

Idiopathic/isolated REM sleep behaviour disorder (iRBD) represents an incipient stage of synucleinopathies. More than 80% of patients are eventually diagnosed with Parkinson’s disease (PD), DLB or multiple system atrophy (MSA).^25^ Systematic evaluation of iRBD cohorts therefore provides an unparalleled opportunity to examine the early prediagnostic course of synucleinopathies directly and assess early biomarkers of PD and DLB.^26-29^ However, it remains unclear why some patients first develop PD and others DLB. Possible mechanisms include differing patterns of synuclein propagation,^30,31^ selective vulnerability related to cortical versus brainstem neurons,^32^ genetic factors, such as *ApoEε4*, and/or AD co-pathology.^33^ Of particular note, early markers of PD and DLB in iRBD are almost identical; olfaction, motor dysfunction, autonomic dysfunction, etc. are highly similar between those who first progress to parkinsonism or dementia.^27^

Plasma amyloid-β and tau biomarkers might be invaluable in patients with iRBD. Detection of AD co-pathology in people with iRBD can have important prognostic implications. Previous work showed that concomitant synuclein and AD pathology is associated with worse global cognition and faster cognitive decline in comparison to people with Lewy body pathology only, also in cognitively unimpaired individuals.^33,34^ Moreover, given that biological and pathophysiological underpinnings of neurodegenerative diseases manifest before clinical signs are observable, plasma markers of AD pathology might create the opportunity to predict risk of future cognitive deterioration long before any cognitive decline is detectable.^1,35,36^ This is especially important in this new era of emerging disease-modifying treatments, because these interventions are hypothesized to be the most effective in the earliest stages of the disease.

We hypothesized that plasma amyloid-β and tau biomarkers might be useful to predict DLB outcome in iRBD. The primary aim of this study was to determine whether plasma amyloid-β and tau biomarkers could predict eventual phenoconversion to DLB in iRBD. Secondary aims were to investigate correlations of these plasma biomarkers with cognitive tests and to determine potential diagnostic accuracy of plasma amyloid-β and tau biomarkers for predicting DLB conversion.

## Materials and methods

### Study design and participants

In this prospective single-centre cohort study, subjects with polysomnography-confirmed iRBD were enrolled prospectively between September 2004 and October 2022 at the Centre for Advanced Research in Sleep Medicine, Hôpital du Sacré-Coeur de Montréal, Canada. Inclusion criteria for the iRBD cohort were age ≥40 years and fulfilment of the clinical and polysomnographic diagnostic criteria for RBD according to the International Classification of Sleep Disorders (third edition). Inclusion criteria for the iRBD cohort were age ≥40 years and fulfilment of the clinical and polysomnographic diagnostic criteria^37^ for RBD according to the International Classification of Sleep Disorders (third edition).^38^ The main exclusion criteria were neurological or neurodegenerative disorders at baseline, i.e. parkinsonism,^39,40^ dementia,^41^ stroke or epilepsy, in addition to epileptiform abnormalities on the EEG. The study was approved by the local ethics committee in accordance with World Medical Association Declaration of Helsinki and Good Clinical Practice guidelines. Written informed consent was obtained from all participants.

All participants were followed annually in order to determine phenoconversion to a defined neurodegenerative disorder (termed ‘phenoconvertors’), based upon meeting diagnostic criteria for parkinsonism (subdivided as PD or MSA according to diagnostic criteria),^39,40^ dementia (note that all dementia phenoconversions by definition meet DLB diagnostic criteria)^41^ or cerebellar subtype of MSA.^40^ The censoring date for phenoconversion status was December 2023. Those not meeting criteria for any of these disorders were considered ‘non-phenoconvertors’. Participants who had no prospective follow-up (minimum follow-up for inclusion = 1year) were excluded, because phenoconversion status was unknown.

### Procedures

#### Plasma sample collection and processing

Blood was collected into EDTA plasma tubes and centrifuged within 30 min of collection at +4°C for 15 min at 1500*g*. Following centrifugation, plasma from all tubes were transferred into one 15 ml polypropylene centrifuge tube and mixed, after which 1.5 ml was aliquoted into 2 ml polypropylene tubes and stored at −8°C within 60 min of collection.

#### Biomarker measurements

All measurements were performed on banked frozen plasma samples, and all samples were measured in singlicate. Plasma samples were analysed with Elecsys® plasma β-Amyloid(1–40), β-Amyloid(1–42) and Elecsys Phospho-Tau(181P) immunoassays as part of NeuroToolKit (NTK, Roche Diagnostics International Ltd), a panel of robust prototype biomarker assays used for research purposes only. Samples were thawed for 30 min at room temperature and roller mixed for 20 min before measuring with Aβ42 and Aβ40 on a cobas® e 601 analyzer (Roche Diagnostics International Ltd). These samples were then transferred directly to cobas® e 801 (Roche Diagnostics International Ltd) to measure pTau181. The NTK assays were validated based on the Guideline on bioanalytical method validation document EMEA/CHMP/EWP/192217/2009 Rev.1 Corr.2 from the European Medicines Agency [Guideline Bioanalytical method validation (europa.eu)].

#### Clinical assessments

Participants underwent annual comprehensive neuropsychological and clinical evaluation by A.P. and R.B.P., as described elsewhere.^42^ Cognitive measures included the following: global cognitive functioning [Montreal Cognitive Assessment (MoCA)^43^]; attention [Trail Making Test (TMT) part A,^44^ Digit Span forward and backward^45^ and Stroop interference^46^]; executive function (Stroop flexibility,^46^ TMT part B,^44^ TMT B minus A,^44^ phonemic^47^ and semantic^48^ verbal fluency); verbal learning and memory (Rey Auditory Verbal Learning Test^49^ -total 1 to 5, -list B, -immediate recall, -delayed recall and -recognition); visuospatial abilities (Rey–Osterrieth Complex Figure Test^50^); and colour vision (Farnsworth–Munsell 100-hue test^51^). MCI was diagnosed using the following criteria: (i) evidence of subjective cognitive complaints by the patient, the spouse or an informant; (ii) evidence of objective cognitive impairment through a neuropsychological assessment; (iii) preservation of daily living functioning; (iv) absence of dementia; and (v) cognitive deficits not solely explained by medication or other medical conditions.^52^  *Z*-scores were calculated as previously described using normative data.^52^  *Z*-scores < −3.5 were set to −3.5 to avoid extreme values.

#### *ApoE* status

*ApoE* status was extracted from imputed genotype data. Genotyping was performed on the OmniExpress GWAS array and NeuroBooster array, according to the manufacturer’s protocols (Illumina Inc.). Quality control of raw genotype data was performed with plink v.1.9 as previously described (https://github.com/neurogenetics/GWAS-pipeline).^53^ Imputation was performed on the cleaned data with the TOPMed Imputation Server using the TOPMed reference panel r3 and default settings.^54,55^

### Statistical analyses

Statistical analyses were performed in R (Rstudio v.2022.07.2, R Foundation, IN, USA) and SPSS (v.29, IBM Corp., NY, USA). The iRBD cohort was stratified for phenoconversion, comparing plasma amyloid-β and pTau181 biomarkers between non-phenoconvertors and DLB convertors, between non-phenoconvertors and PD convertors, and between non-phenoconvertors and all phenoconvertors. In secondary subgroup analyses, we also assessed plasma markers among PD phenoconvertors stratified to presence of MCI at the time of phenoconversion. One additional *post hoc* exploratory analysis was conducted, dividing the dataset based on normal MoCA (score of ≥26) versus abnormal MoCA (score of ≤25) at the time of blood sampling. This stratification aimed to explore differences between non-phenoconvertors and DLB convertors within both normal and abnormal MoCA score ranges. Finally, biomarker results were stratified based on *ApoE* genotype (*ApoEε4* non-carriers versus carriers) to explore whether plasma marker levels differed between iRBD patients carrying the *ApoEε4* allele and *ApoEε4* non-carriers. Distribution of data was assessed using histograms and QQ-plots. One-way ANOVA adjusted for age and sex was performed for all group analyses. If significant, Dunnett’s test to contrast phenoconvertors with non-phenoconvertors was performed. Unadjusted Cohen’s *d* effect sizes were calculated; effect amplitude was described according to convention as small (*d* > 0.2), moderate (*d* > 0.5) or large (*d* > 0.8).

Receiver operating characteristic (ROC) curve analysis was used to determine diagnostic accuracy of Aβ42/40 and pTau181. Optimal thresholds for each marker were determined by the Youden index (i.e. value that maximized combination of sensitivity and specificity). For this analysis, selecting a suitable reference group was challenging, because no healthy control group was available, and many iRBD non-phenoconvertors will later develop DLB (artificially decreasing specificity estimates). To minimize the number of early DLB subjects in the reference group and considering that MCI is a risk factor for DLB, we selected only those iRBD non-phenoconvertors who remained free of MCI at the most recent visit.

Time-to-event analysis using the Kaplan–Meier estimator was performed after stratification by the optimal Youden index-based threshold for Aβ42/40 and pTau181. The log-rank method was used to estimate significance of differences in event rates between biomarker groups, and hazard ratios were calculated using Cox proportional hazards analysis. To ensure that results were not overfitted, we repeated the time-to-event analysis with a data-driven approach, using stratification based on quantiles of biomarker levels rather than the optimal binary cut-off.

Linear regression adjusted for age and sex was performed to interrogate associations between cognitive tests and plasma biomarkers for the total iRBD cohort.

Two-tailed *P*-values < 0.05 were considered significant. Primary outcomes involved plasma Aβ42/40 ratio and pTau181 in DLB convertors. The *P*-values are reported with false discovery rate correction using the Benjamini–Yekutieli procedure. Other outcomes were secondary and considered exploratory. Results are shown as the mean ± standard deviation (SD), unless otherwise specified.

## Results

### Participants

A total of 158 iRBD participants consented to blood sampling. Of these, 14 were excluded: six owing to loss to follow-up (Supplementary Table 1) and eight because blood sampling was done during the same visit when they were diagnosed with PD (*n* = 6) or DLB (*n* = 2) (hence they could no longer be considered iRBD at time of sampling). Of the remaining 144 participants, 32 eventually phenoconverted to a defined neurodegenerative disorder at the censoring date (December 2023). Finally, two biomarker results were excluded as outliers (>4 SD from the mean). Therefore, plasma biomarkers were available in 110 non-phenoconvertors, 18 DLB convertors, 13 PD convertors and one MSA convertor. (Supplementary Fig. 1) Demographic characteristics are summarized in Table 1. Phenoconvertors were slightly older than non-phenoconvertors (70.6 versus 66.7 years, *P* = 0.01), particularly for DLB convertors (73.3 years, *P* = 0.001). Time between blood sampling and disease diagnosis was 2.9 years for phenoconvertors. The time between blood sampling and last visit for non-phenoconvertors was 4.4 years. Cognitive scores at baseline are summarized in Supplementary Table 2.

**Table 1:** Demographic characteristics at time of blood sampling

Characteristic	iRBD non-phenoconvertors	iRBD phenoconvertors^a^	*P*-value^b^	DLB convertors	*P*-value^c^	PD convertors	*P*-value^c^
Number	110	32	–	18	–	13	–
Age, mean ± SD, years	66.7 ± 8.1	70.6 ± 7.1	0.01	73.3 ± 7.2	0.001	68.2 ± 3.8	0.67
Sex (male/female)	82/28	27/5	0.36	14/4	0.99	12/1	0.30
Education, mean ± SD, years	14.4 ± 3.7	14.2 ± 3.5	0.81	12.8 ± 3.6	0.14	16.0 ± 2.5	0.08
Follow-up after blood sample, mean ± SD, years	4.4 ± 2.2	2.9 ± 2.1	<0.001	2.4 ± 1.7	<0.001	3.3 ± 2.4	0.02

Data are shown as the mean ± standard deviation (SD). DLB = dementia with Lewy bodies; iRBD = idiopathic/isolated REM sleep behaviour disorder; PD = Parkinson’s disease.

^a^No separate subgroup column was created for the single multiple system atrophy convertor.

^b^ *P*-values show comparison between iRBD without and with phenoconversion during follow-up with Student’s *t*-test or χ^2^ test.

^c^ *P*-values show comparison between non-phenoconvertors and DLB convertors, or non-phenoconvertors and PD convertors with Mann–Whitney U-test or Fisher’s exact test.

### Plasma biomarkers as predictors of phenoconversion

**Figure 1:**
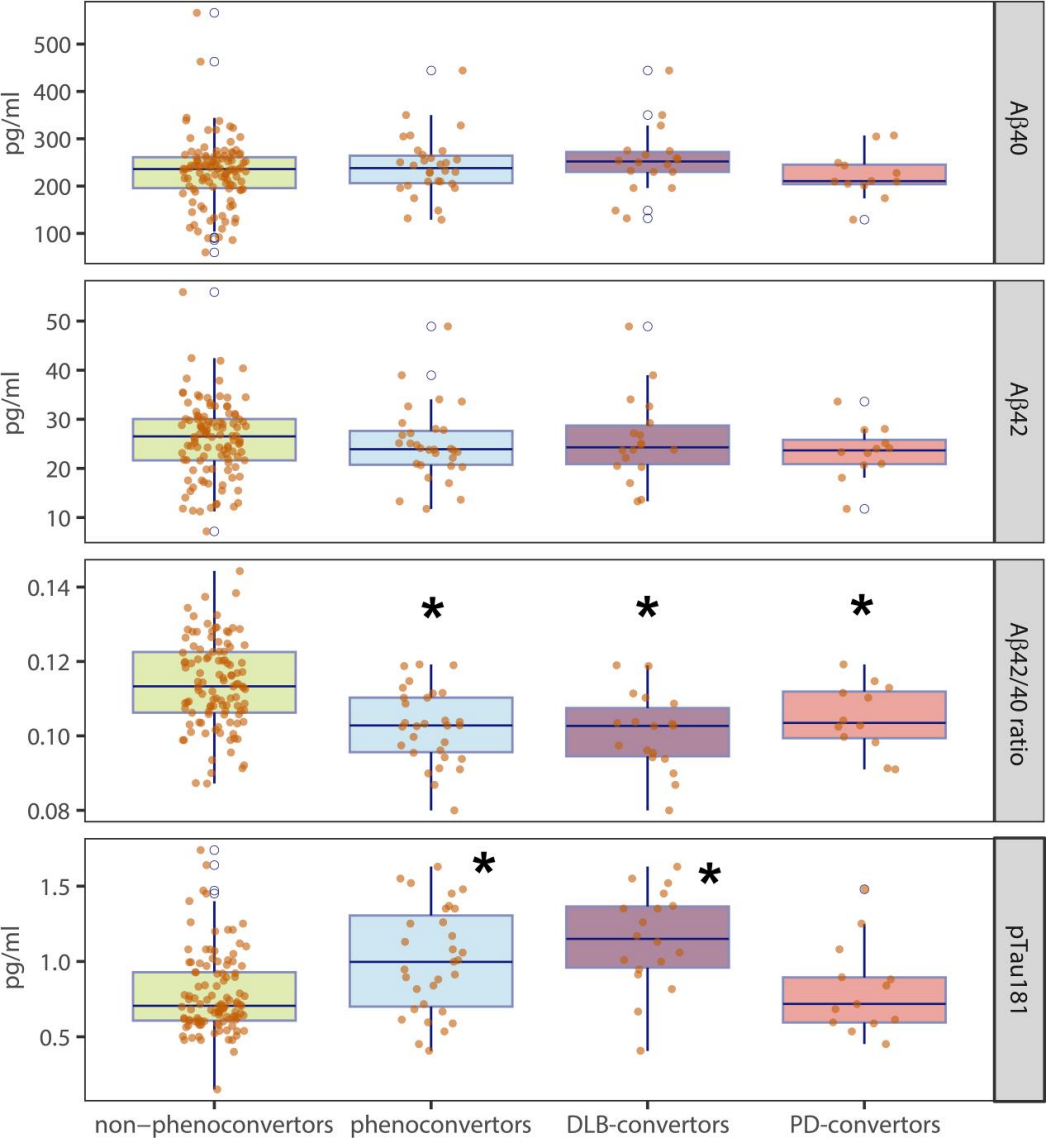
**Plasma biomarker levels in the iRBD cohort.** Box plots and individual levels for different plasma Alzheimer's disease-related biomarkers in iRBD for non-phenoconvertors compared to all phenoconvertors, as well as to DLB and PD convertor groups separately. *Significantly different from non-phenoconvertors. DLB = dementia with Lewy bodies; iRBD = idiopathic/isolated REM sleep behaviour disorder; PD = Parkinson’s disease; pTau181 = tau phosphorylated at threonine 181.

In comparison to non-phenoconvertors, DLB convertors had significantly lower plasma Aβ42/40 at baseline [difference −0.011, 95% confidence interval (CI) (−0.016; −0.005), *P* < 0.001]. Baseline pTau181 was significantly higher in those destined to develop DLB than in non-phenoconvertors [difference 0.282 pg/ml, 95% CI (0.146; 0.418), *P* < 0.001]. The effect size for DLB convertors was large in both comparisons, with Cohen’s *d* = 1.31 for pTau181 and *d* = 1.12 for Aβ42/40 (Table 2 and Fig. 1).

**Table 2:** Summary of plasma amyloid-β and pTau181 biomarkers in iRBD cohort stratified by phenoconversion status

Plasma biomarker	iRBD non-phenoconvertors *n* = 110	iRBD phenoconvertors *n* = 32	*P*-value^a^	Cohen’s *d*^c^	DLB convertors *n* = 18	PD convertors *n* = 13	*P*-value^b^	Dunnett’s *post hoc P*-value^b^		Cohen’s *d*^c^	
								DLB	PD	DLB	PD
Aβ40, mean ± SD, pg/ml	228.3 ± 70.8	235.1 ± 72.1	0.65	0.10	253.7 ± 71.3	222.8 ± 50.0	0.74	NA	NA	0.36	0.08
Aβ42, mean ± SD, pg/ml	25.82 ± 7.77	24.27 ± 8.20	0.10	0.20	25.87 ± 8.77	23.39 ± 5.45	0.39	NA	NA	0.01	0.32
Aβ42/40 ratio, mean ± SD	0.114 ± 0.012	0.103 ± 0.010	0.0003*	0.94	0.101 ± 0.010	0.105 ± 0.009	0.0006*	0.0008*	0.036*	1.12	0.79
pTau181, mean ± SD, pg/ml	0.784 ± 0.266	0.993 ± 0.354	0.008*	0.73	1.144 ± 0.326	0.816 ± 0.302	0.0006*	0.0002*	0.97	1.31	0.12

Data are shown as the mean ± standard deviation (SD) for the different plasma biomarkers in non-phenoconvertors, all phenoconvertors and in the DLB and PD convertor subgroups. Phenoconversion status was based on the disease status at the time of analysis. DLB = dementia with Lewy bodies; iRBD = idiopathic/isolated REM sleep behaviour disorder; NA = not applicable; PD = Parkinson’s disease; pTau181 = tau phosphorylated at threonine 181.

^*^Significant *P*-values.

^a^One-way ANOVA adjusted for age and sex was used for group comparisons with *post hoc* false discovery rate correction using the Benjamini–Yekutieli procedure for primary outcomes Aβ42/40 ratio and pTau181.

^b^One-way ANOVA adjusted for age and sex was used for group comparisons with *post hoc* false discovery rate correction using the Benjamini–Yekutieli procedure for primary outcomes Aβ42/40 ratio and pTau181, followed by Dunnett’s *post hoc* test for the subgroup analysis (non-phenoconvertors versus DLB convertors and non-phenoconvertors versus PD convertors).

^c^Cohen’s *d* effect size unadjusted for age and sex.

When comparing non-phenoconvertors with PD convertors, differences were less clear. Plasma Aβ42/40 was lower in PD convertors [difference −0.008, 95% CI (−0.014; −0.001), *P* = 0.036, Cohen’s *d* = 0.79], but no significant differences were found for pTau181 (Table 2 and Fig. 1). When stratified according to MCI status, plasma Aβ42/40 in PD convertors with MCI was lower than in non-phenoconvertors [difference −0.011, 95% CI (−0.019; −0.003), *P* = 0.03, Cohen’s *d* = 1.04] with biomarker profiles resembling those of DLB convertors, but again pTau181 levels were not significantly different [difference 0.119 pg/ml, 95% CI (−0.070; 0.308), *P* = 0.46, Cohen’s *d* = 0.49]. In contrast, in PD convertors without MCI, both plasma Aβ42/40 and pTau181 were similar to non-phenoconvertors (Supplementary Fig. 2).

When comparing non-phenoconvertors with all phenoconvertors (i.e. DLB, PD and MSA combined), Aβ42/40 was lower in phenoconvertors [difference −0.009, 95% CI (−0.013; −0.004), *P* < 0.001, Cohen’s *d* = 0.94], and pTau181 was higher [difference 0.157 pg/ml, 95% CI (0.049; 0.266), *P* = 0.008, Cohen’s *d* = 0.73] (Table 2 and Fig. 1). pTau181 was moderately correlated with Aβ40/Aβ42 (*r* = −0.33, *P* < 0.001) (Supplementary Fig. 3).

Finally, we stratified the dataset based on normal MoCA versus abnormal MoCA score (Supplementary Fig. 4 and Supplementary Table 3). This showed the same pattern of lower plasma Aβ42/40 and higher pTau181 levels in DLB convertors versus non-phenoconvertors for normal and abnormal MoCA score subgroups.

### Accuracy of plasma biomarkers for DLB prediction

Detailed results of ROC curve analysis are shown in Supplementary Table 4 and Supplementary Fig. 5. Aβ42/40 showed an area under the curve of 0.84. The optimal Aβ42/40 threshold was 0.112, which provided 89% sensitivity with 63% specificity, a positive predictive value of 35% and negative predictive value of 96% (note again that specificity is likely to be underestimated because the reference group was iRBD non-phenoconvertors, of whom many will develop DLB). pTau181 showed an area under the curve of 0.85. The optimal pTau181 threshold to identify DLB convertors was 0.802 pg/ml, resulting in 89% sensitivity and 73% specificity, with 42% positive predictive value and 97% negative predictive value. For DLB versus PD convertors, ROC curve analysis demonstrated lower area under the curve [0.62 for Aβ42/40, 95% CI (0.41; 0.83); 0.78 for pTau181, 95% CI (0.61; 0.96)] than ROC curve analysis for DLB convertors versus non-phenoconvertors.

### Time-to-event analysis

**Figure 2:**
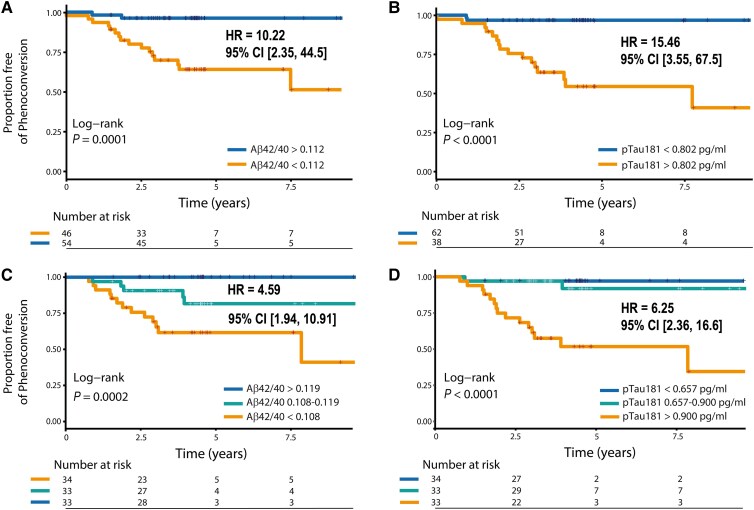
**Kaplan–Meier curves based on optimal thresholds or quantiles for different markers.** Kaplan–Meier time-to-event curves for plasma Aβ42/40 ratio (**A** and **C**) and pTau181 (**B** and **D**) for DLB phenoconversion (event) using non-phenoconvertors without mild cognitive impairment as reference, stratified based on the optimal Youden index -based threshold for both markers (**A** and **B**) or based on three-quantiles (**C** and **D**). CI = confidence interval; DLB = dementia with Lewy bodies; HR = hazard ratio; pTau181 = tau phosphorylated at threonine 181.

Kaplan–Meier curves are shown in Fig. 2. Stratification based on the optimal cut-off (Aβ42/40 < 0.112 and pTau181 > 0.802 pg/ml) resulted in a hazard ratio for phenoconversion to DLB of 10.2 for Aβ42/40 [95% CI (2.3; 44.5), *P* = 0.003] and 15.5 for pTau181 [95% CI (3.5; 67.5), *P* < 0.0001].

Data-drive time-to-event analysis based on quantiles of the biomarker levels largely replicated the results using binarization based on the optimal threshold (Fig. 2 and Supplementary Table 5). The quantiles with the highest pTau181 levels consistently showed a significantly higher number of events (i.e. DLB diagnosis) compared with the lower quantiles. In our dataset, cut-offs of pTau181 > 0.900 pg/ml seemed to be associated with a risk of future DLB diagnosis in iRBD, whereas pTau181 < 0.718 pg/ml appeared to predict a low likelihood of DLB phenoconversion. Similar results were found for plasma Aβ42/40, with the distinction of DLB risk based on baseline Aβ42/40 ratio, i.e. the lowest Aβ42/40 quantile showed the highest number of events. This suggested that Aβ42/40 < 0.108 was associated with DLB phenoconversion, whereas Aβ42/40 > 0.119 seemed to be associated with a lower risk of future DLB diagnosis in our iRBD cohort. For both pTau181 and Aβ42/40, associated hazard ratios were still considerable and reflect a more generalizable value in comparison to the binary approach using the optimal cut-off (Fig. 2 and Supplementary Table 5).

### Correlation of plasma amyloid-β and pTau181 with cognitive scores in iRBD

**Figure 3:**
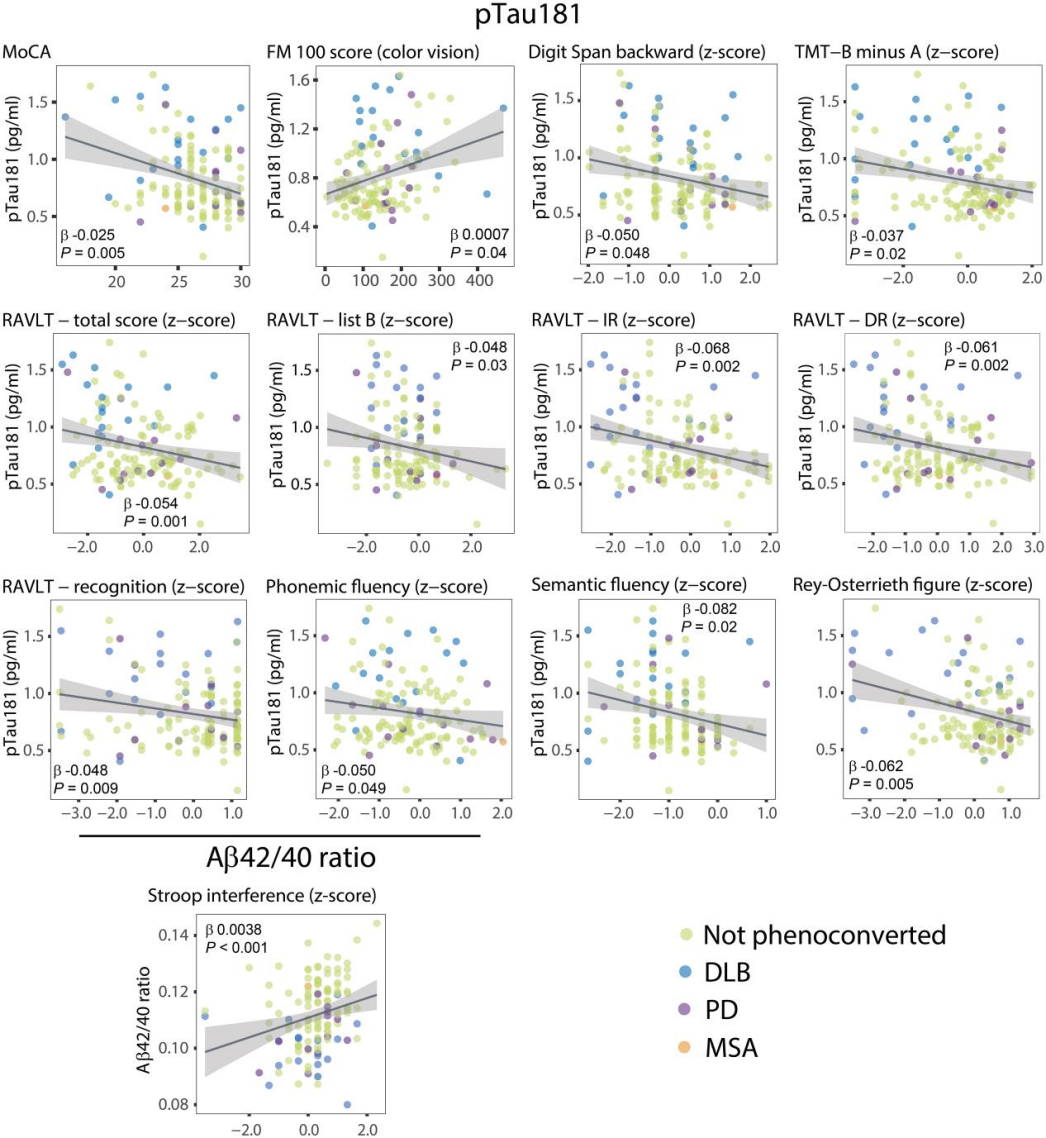
**Associations between plasma biomarkers and cognitive testing in iRBD.** Scatter plot and regression fit line showing the significant correlations between pTau181 levels or Aβ42/40 ratio and different cognitive tests in the iRBD cohort. Linear regression models are adjusted for age and sex. Individual data-points are coloured according to iRBD subgroup. The shaded band of the regression line represents the 95% confidence interval. DLB = dementia with Lewy bodies; FM-100 = Farnsworth-Munsell 100-hue test; iRBD = idiopathic/isolated REM sleep behaviour disorder; MoCA = Montreal Cognitive Assessment; MSA = multiple system atrophy; PD = Parkinson's disease; pTau181 = tau phosphorylated at threonine 181; RAVLT = Rey Auditory Verbal Learning Test; RAVLT-DR = delayed recall; RAVLT-IR = immediate recall; RAVLT-total score = trials 1–5; Rey–Osterrieth figure = Rey-Osterrieth Complex Figure Test; TMT = Trail Making Test.

We interrogated cross-sectional correlations between plasma biomarkers and cognitive tests in different domains and in colour vision perception,^23^ a well-studied marker of early DLB (Fig. 3 and Supplementary Table 6). In general, cognitive testing correlations were stronger for pTau181 than for Aβ42/40. Global cognitive screening with MoCA was correlated with pTau181 (β = −0.025, *P* = 0.005). Stroop interference was correlated with Aβ42/40 (β = 0.0038, *P* < 0.001). pTau181 was also correlated with measures of attention and executive function including Digit Span backwards (β = −0.050, *P* = 0.048), semantic (β = −0.082, *P* = 0.02) and phonemic (β = −0.050, *P* = 0.049) verbal fluency, and TMT B minus A (β = −0.037, *P* = 0.02). Likewise, tests of verbal memory were correlated with plasma pTau181, including learning (Rey Auditory Verbal Learning Test-total, β = −0.054, *P* = 0.001), immediate recall (β = −0.068, *P* = 0.002), delayed recall (β = −0.061, *P* = 0.002) and recognition (β = −0.048, *P* = 0.009). Finally, pTau181 showed correlations with visuospatial function assessed with Rey–Osterrieth Complex Figure Test (β = −0.062, *P* = 0.005) and with colour vision (β = 0.0007, *P* = 0.04).

### Stratification based on *ApoE* status

*ApoE* genotype was available for 106 participants, of whom 20 were heterozygous for the *ApoEε4* allele and one was homozygous. *ApoEε4* carriers showed significantly lower Aβ42 [difference = −4.88 pg/ml, 95% CI (−8.55; −1.20), *P* = 0.01] and Aβ42/40 [difference = −0.009, 95% CI (−0.014; −0.004), *P* < 0.001]. Effect sizes were moderate for both markers (Cohen’s *d* Aβ42 = 0.71; Aβ42/40 = 0.70). There were no differences for pTau181 (Supplementary Table 7 and Supplementary Fig. 6).

## Discussion

The primary finding of this study is that blood Aβ42/40 and pTau181 can predict development of DLB in iRBD. This is true for both biomarkers, but pTau181 seems particularly useful for distinguishing DLB convertors from non-phenoconvertors. Even when using a comparison group at risk of DLB, ROC curve analysis revealed relatively high sensitivity and specificity of these biomarkers. From a mechanistic perspective, this suggests that within iRBD, a disorder generally defined as a synucleinopathy, AD pathology might be one of the drivers of eventual clinical phenoconversion subtype.

Although CSF AD biomarkers are well established, plasma biomarkers have only been described recently and are undergoing standardization.^4,8,11^ Blood-based biomarkers have the major advantage of being more accessible than CSF or PET imaging.^4^ Within AD itself, plasma biomarkers of AD pathology are strongly correlated with CSF biomarkers and with amyloid/tau PET imaging.^9,11,56,57^ However, plasma AD markers in DLB have been less well studied. Previous work in manifest DLB showed higher plasma pTau181 and pTau231 levels compared with healthy controls, but lower levels compared with AD and no difference from PD.^18,19,21,22^ For plasma pTau181, a cross-sectional study in MCI-LB showed higher levels than controls, albeit with levels lower than MCI-AD, whereas another study did not find differences between MCI-LB and controls.^19,20^ Our study shows that these biomarkers, beyond identifying prevalent DLB, also can predict DLB outcome in a condition that identifies incipient synucleinopathy. Moreover, the absence of these markers suggests that DLB phenoconversion in iRBD over the next 4–5 years is unlikely.

The NTK toolkit is an ongoing field of research, and validated reference values for plasma pTau181 and Aβ42/40 levels in healthy older adults and/or AD are not yet available, nor are cut-off values for abnormal versus normal levels. Cut-off values would allow more accurate determination of whether iRBD patients (and particularly DLB convertors) have pathological levels of plasma amyloid-β and pTau markers, facilitating the interpretation of these markers and enhancing the predictive value of these markers in iRBD. Nonetheless, given that this represents the first study using the NTK in iRBD and in synucleinopathies in general, there are no previous data or results for reference or comparison. Comparison of our findings with a recent study in the BioFINDER cohort, also using Elecsys plasma immunoassays on cobas® e 411 and cobas® e 601 (Roche Diagnostics) for plasma Aβ40, Aβ42 and pTau181 measurements, revealed that the mean plasma Aβ42/40 in early DLB seems to align with individuals having CSF Aβ-positive status; for plasma pTau181, the mean levels in our DLB convertors appear to be intermediate between those observed in individuals with CSF Aβ-positive versus negative status.^11^

The cross-sectional correlation analysis yielded some notable observations. In general, higher plasma pTau181 was associated with poorer global cognitive functioning and cognitive domains, including attention/executive, verbal learning and visuospatial. In contrast, only one measure of attention was correlated with Aβ42/40. Similar correlations in manifest DLB have been found between Mini-Mental State Examination and plasma pTau, although a study in MCI-LB found no correlation.^18,20^ For Aβ42/40, a prior study in DLB reported correlation with 1-year Mini-Mental State Examination decline, whereas another study failed to find such correlation.^16,58^ Of note, in prior prospective studies of cognitive dysfunction in iRBD, dysfunction in the attention/executive/verbal learning domains that best identified early DLB were the same as those correlating with pTau181 levels in our present study.^26,59^ Taken together, these findings suggest that while plasma Aβ42/40 might suggest AD pathology in iRBD, pTau181 was the plasma marker most correlated with cognitive tests. This corroborates with recent studies showing a correlation of plasma pTau181, and not Aβ42/40, with cognitive impairment in DLB.^21,22^ It is also consistent with AD staging models positing that amyloid changes are early events, whereas later-stage tau deposition is correlated best with neurodegeneration and its subsequent clinical phenotype.^60^ Our findings might suggest potential for pTau181 as an exploratory end point in clinical trials, although this would require further validation on larger samples and longitudinal data.

*ApoEε4* has been linked to dementia in synucleinopathies.^61^ In the present study, *ApoEε4* carriers showed lower plasma Aβ42 levels and Aβ42/40 ratio than *ApoEε4* non-carriers, with no differences in plasma pTau181 levels. Lower CSF Aβ42 levels in *ApoEε4* carriers have previously been found in AD^62^ and DLB^63^ cohorts. The effect of *ApoEε4* genotype observed in our study is therefore consistent with mounting evidence that *ApoEε4* is linked to higher levels of AD co-pathology within DLB,^11,62,63^ here suggested even in its early stages.

iRBD is the strongest known early marker of neurodegenerative synucleinopathy; 98% of polysomnography-proven iRBD cases have synuclein deposition on autopsy,^64^ and >80% of typical late-onset iRBD cases eventually develop a defined neurodegenerative synucleinopathy condition.^25^ However, to this date it had remained unclear why some patients develop parkinsonism first whereas others develop dementia first. Of note, clinical predictive markers in iRBD are almost indistinguishable between PD and DLB phenoconvertors, with similar levels of autonomic dysfunction, mood changes, olfactory loss and motor changes (quantitative motor testing is an even earlier predictor of DLB than PD).^26,27,29^ The only markers that have been able to distinguish outcome are cognitive parameters and colour vision testing (the latter is also considered a perceptual cognitive task).^26,27,29^ Numerous mechanistic hypotheses for this different outcome can be posited, including different patterns of synuclein spread^30,31^ (perhaps attributable to differences in synuclein polymorphisms^65^ and/or different intrinsic structure of synuclein fibrils^66^), variable selective vulnerability related to intrinsic factors of substantia nigral versus cortical neurons to synuclein-mediated degeneration,^32^ genetic factors (such as *ApoEε4* status, associated with more severe Lewy body pathology^61^ and with dementia in PD) or co-morbid pathology (e.g. AD pathology).^14,33^ The present findings support the latter hypothesis, namely that comorbid AD pathology might serve as a potential critical driver of the differential clinical fate of certain iRBD patients.

A recent study reported plasma biomarkers in the DLB continuum, including 15 patients with iRBD.^19^ No differences for Aβ42/40 were found between controls and iRBD, MCI-LB or DLB. pTau181 levels were increased in DLB compared with controls, but no differences were found for MCI-LB or iRBD.^19^ That study investigated cross-sectional group differences, in contrast to the prospective outcome of phenoconversion in iRBD in our study.

Some limitations should be noted. Clinical disease outcome was classified based on diagnostic criteria for DLB,^41^ PD^39^ or MSA^40^; it is possible that these diagnoses might have been incorrect in some cases. Although our sample was large in comparison to typical iRBD cohorts, the number of individuals who phenoconverted to each neurodegenerative disorder was relatively small, hence estimates of sensitivity and specificity might be imprecise. Average follow-up duration is short, which might underestimate the results, because more phenoconvertors are expected with longer follow-up. Owing to the exploratory nature of this study and the lack of direct reference values in healthy controls, no cut-off values for normal versus abnormal levels in people with iRBD were available or could be determined. More data and research using the NTK toolkit in early synucleinopathies are needed, also including head-to-head comparisons with plasma levels in pathologically proven AD and/or healthy older adults. Further studies in larger cohorts of early synucleinopathies are needed to replicate these findings, also including healthy control data, in addition to longitudinal assessment of changes in plasma biomarker levels over time. Despite significant group differences and a decent effect size at the group level, the absolute difference in plasma pTau181 and Aβ42/40 levels and the fold-change, especially for Aβ42/40 in the DLB convertor group, is rather low, which may be a challenge for its clinical applicability. Next, we measured plasma levels of pTau181, because the alternative tau markers, pTau217 and pTau231, were not available yet. In particular, plasma pTau181 might not be specific to AD pathology, given that elevations have been found in neuromuscular diseases such as amyotrophic lateral sclerosis and spinal muscular atrophy.^23,24^ No CSF-based markers for AD pathology or amyloid PET imaging were available in our cohort; these would be important to confirm the specificity of our findings for AD-related pathology. In comparison to plasma pTau181, plasma pTau231 might be more sensitive to the earliest AD stages,^8^ and pTau217 might have the highest accuracy to discriminate AD from other neurodegenerative disorders and track longitudinal increases with disease progression.^56^ Future studies need to assess the diagnostic value of pTau217 and pTau231 in iRBD. Finally, in interpreting our ROC analysis, it is essential to note that the reference group (i.e. non-DLB) were participants with iRBD, most of whom will eventually phenoconvert to DLB or another synucleinopathy. Observations from our cohort have shown that of those participants with iRBD free of MCI at baseline, 8% will develop DLB over the following 3 years (data not shown). If these eventual DLB phenoconvertors would already have abnormal plasma AD biomarkers, these would incorrectly appear as ‘false positives’ in ROC analysis, artificially driving lower specificity and positive predictive value. Therefore, true specificity and positive predictive value might be higher than we estimated.

In contrast, strengths of our study are the prospective dataset of subjects on a relatively large group of patients with incipient synucleinopathy, followed annually after blood sampling for phenoconversion to defined neurodegenerative disease. All cases of iRBD were confirmed by polysomnography. Comprehensive clinical assessments were available for all participants.

## Conclusion

In conclusion, our findings suggest that the plasma Aβ42/40 ratio and pTau181 can predict DLB outcome in iRBD. In certain iRBD patients, plasma markers of amyloid-β and/or tau can predict whether patients will ultimately develop primary dementia or parkinsonism.

## Supplementary Material

awaf003_Supplementary_Data

## Data Availability

Data that support the findings of this study are available on reasonable request from the corresponding author, respecting patient informed consent and ethical or legal restrictions.

## References

[1] Roberts RO, Aakre JA, Kremers WK, et al Prevalence and outcomes of amyloid positivity among persons without dementia in a longitudinal, population-based setting. JAMA Neurol. 2018;75:970–979. 29710225 10.1001/jamaneurol.2018.0629 PMC6142936

[2] Dubois B, von Arnim CAF, Burnie N, Bozeat S, Cummings J. Biomarkers in Alzheimer’s disease: Role in early and differential diagnosis and recognition of atypical variants. Alzheimers Res Ther. 2023;15:175. 37833762 10.1186/s13195-023-01314-6 PMC10571241

[3] Schoonenboom NS, Reesink FE, Verwey NA, et al Cerebrospinal fluid markers for differential dementia diagnosis in a large memory clinic cohort. Neurology. 2012;78:47–54. 22170879 10.1212/WNL.0b013e31823ed0f0

[4] Leuzy A, Mattsson-Carlgren N, Palmqvist S, Janelidze S, Dage JL, Hansson O. Blood-based biomarkers for Alzheimer’s disease. EMBO Mol Med. 2022;14:e14408. 34859598 10.15252/emmm.202114408 PMC8749476

[5] Janelidze S, Mattsson N, Palmqvist S, et al Plasma P-tau181 in Alzheimer’s disease: Relationship to other biomarkers, differential diagnosis, neuropathology and longitudinal progression to Alzheimer’s dementia. Nat Med. 2020;26:379–386. 32123385 10.1038/s41591-020-0755-1

[6] Palmqvist S, Janelidze S, Quiroz YT, et al Discriminative accuracy of plasma phospho-tau217 for Alzheimer disease vs other neurodegenerative disorders. JAMA. 2020;324:772–781. 32722745 10.1001/jama.2020.12134 PMC7388060

[7] Thijssen EH, La Joie R, Strom A, et al Plasma phosphorylated tau 217 and phosphorylated tau 181 as biomarkers in Alzheimer’s disease and frontotemporal lobar degeneration: A retrospective diagnostic performance study. Lancet Neurol. 2021;20:739–752. 34418401 10.1016/S1474-4422(21)00214-3 PMC8711249

[8] Ashton NJ, Janelidze S, Mattsson-Carlgren N, et al Differential roles of Aβ42/40, p-tau231 and p-tau217 for Alzheimer’s trial selection and disease monitoring. Nat Med. 2022;28:2555–2562. 36456833 10.1038/s41591-022-02074-w PMC9800279

[9] Mielke MM, Hagen CE, Xu J, et al Plasma phospho-tau181 increases with Alzheimer’s disease clinical severity and is associated with tau- and amyloid-positron emission tomography. Alzheimers Dement. 2018;14:989–997. 29626426 10.1016/j.jalz.2018.02.013 PMC6097897

[10] Verberk IMW, Hendriksen HMA, van Harten AC, et al Plasma amyloid is associated with the rate of cognitive decline in cognitively normal elderly: The SCIENCe project. Neurobiol Aging. 2020;89:99–107. 32081465 10.1016/j.neurobiolaging.2020.01.007

[11] Palmqvist S, Stomrud E, Cullen N, et al An accurate fully automated panel of plasma biomarkers for Alzheimer’s disease. Alzheimers Dement. 2023;19:1204–1215. 35950735 10.1002/alz.12751 PMC9918613

[12] Cullen NC, Leuzy A, Janelidze S, et al Plasma biomarkers of Alzheimer’s disease improve prediction of cognitive decline in cognitively unimpaired elderly populations. Nat Commun. 2021;12:3555. 34117234 10.1038/s41467-021-23746-0 PMC8196018

[13] Dugger BN, Adler CH, Shill HA, et al Concomitant pathologies among a spectrum of parkinsonian disorders. Parkinsonism Relat Disord. 2014;20:525–529. 24637124 10.1016/j.parkreldis.2014.02.012 PMC4028418

[14] Howlett DR, Whitfield D, Johnson M, et al Regional multiple pathology scores are associated with cognitive decline in Lewy body dementias. Brain Pathol. 2015;25:401–408. 25103200 10.1111/bpa.12182 PMC8029273

[15] Jain L, Khrestian M, Formica S, et al ATN cerebrospinal fluid biomarkers in dementia with Lewy bodies: Initial results from the United States Dementia with Lewy Bodies Consortium. Alzheimers Dement. 2024;20:549–562. 37740924 10.1002/alz.13398 PMC10840643

[16] van Steenoven I, van der Flier WM, Scheltens P, Teunissen CE, Lemstra AW. Amyloid-β peptides in cerebrospinal fluid of patients with dementia with Lewy bodies. Alzheimers Res Ther. 2019;11:83. 31601267 10.1186/s13195-019-0537-5 PMC6788069

[17] Chouliaras L, Thomas A, Malpetti M, et al Differential levels of plasma biomarkers of neurodegeneration in Lewy body dementia, Alzheimer’s disease, frontotemporal dementia and progressive supranuclear palsy. J Neurol Neurosurg Psychiatry. 2022;93:651–658. 35078917 10.1136/jnnp-2021-327788 PMC9148982

[18] Gonzalez MC, Ashton NJ, Gomes BF, et al Association of plasma p-tau181 and p-tau231 concentrations with cognitive decline in patients with probable dementia with Lewy bodies. JAMA Neurol. 2022;79:32–37. 34807233 10.1001/jamaneurol.2021.4222 PMC8609462

[19] Diaz-Galvan P, Przybelski SA, Algeciras-Schimnich A, et al Plasma biomarkers of Alzheimer’s disease in the continuum of dementia with Lewy bodies. Alzheimers Dement. 2024;20:2485–2496. 38329197 10.1002/alz.13653 PMC11032523

[20] Thomas AJ, Hamilton CA, Heslegrave A, et al A longitudinal study of plasma pTau181 in mild cognitive impairment with Lewy bodies and Alzheimer’s disease. Mov Disord. 2022;37:1495–1504. 35318733 10.1002/mds.28994 PMC9540809

[21] Vrillon A, Bousiges O, Gotze K, et al Plasma biomarkers of amyloid, tau, axonal, and neuroinflammation pathologies in dementia with Lewy bodies. Alzheimers Res Ther. 2024;16:146. 38961441 10.1186/s13195-024-01502-y PMC11221164

[22] Bolsewig K, van Unnik A, Blujdea ER, et al Association of plasma amyloid, P-tau, GFAP, and NfL with CSF, clinical, and cognitive features in patients with dementia with Lewy bodies. Neurology. 2024;102:e209418. 38830138 10.1212/WNL.0000000000209418 PMC11244745

[23] Vacchiano V, Mastrangelo A, Zenesini C, et al Elevated plasma p-tau181 levels unrelated to Alzheimer’s disease pathology in amyotrophic lateral sclerosis. J Neurol Neurosurg Psychiatry. 2023;94:428–435. 37012065 10.1136/jnnp-2022-330709

[24] Cousins KAQ, Shaw LM, Shellikeri S, et al Elevated plasma phosphorylated tau 181 in amyotrophic lateral sclerosis. Ann Neurol. 2022;92:807–818. 35877814 10.1002/ana.26462 PMC9588516

[25] Postuma RB, Iranzo A, Hu M, et al Risk and predictors of dementia and parkinsonism in idiopathic REM sleep behaviour disorder: A multicentre study. Brain. 2019;142:744–759. 30789229 10.1093/brain/awz030 PMC6391615

[26] Joza S, Hu MT, Jung KY, et al Prodromal dementia with Lewy bodies in REM sleep behavior disorder: A multicenter study. Alzheimers Dement. 2024;20:91–102. 37461299 10.1002/alz.13386 PMC10917000

[27] Fereshtehnejad SM, Yao C, Pelletier A, Montplaisir JY, Gagnon JF, Postuma RB. Evolution of prodromal Parkinson’s disease and dementia with Lewy bodies: A prospective study. Brain. 2019;142:2051–2067. 31111143 10.1093/brain/awz111

[28] Siderowf A, Concha-Marambio L, Lafontant DE, et al Assessment of heterogeneity among participants in the Parkinson’s Progression Markers Initiative cohort using α-synuclein seed amplification: A cross-sectional study. Lancet Neurol. 2023;22:407–417. 37059509 10.1016/S1474-4422(23)00109-6 PMC10627170

[29] Joza S, Hu MT, Jung KY, et al Progression of clinical markers in prodromal Parkinson’s disease and dementia with Lewy bodies: A multicentre study. Brain. 2023;146:3258–3272. 36881989 10.1093/brain/awad072

[30] Borghammer P, Horsager J, Andersen K, et al Neuropathological evidence of body-first vs. brain-first Lewy body disease. Neurobiol Dis. 2021;161:105557. 34763110 10.1016/j.nbd.2021.105557

[31] Adler CH, Beach TG. Neuropathological basis of nonmotor manifestations of Parkinson’s disease. Mov Disord. 2016;31:1114–1119. 27030013 10.1002/mds.26605 PMC4981515

[32] Haddad D, Nakamura K. Understanding the susceptibility of dopamine neurons to mitochondrial stressors in Parkinson’s disease. FEBS Lett. 2015;589(24 Pt A):3702–3713. 26526613 10.1016/j.febslet.2015.10.021 PMC4679488

[33] Palmqvist S, Rossi M, Hall S, et al Cognitive effects of Lewy body pathology in clinically unimpaired individuals. Nat Med. 2023;29:1971–1978. 37464059 10.1038/s41591-023-02450-0 PMC10427420

[34] Quadalti C, Palmqvist S, Hall S, et al Clinical effects of Lewy body pathology in cognitively impaired individuals. Nat Med. 2023;29:1964–1970. 37464058 10.1038/s41591-023-02449-7 PMC10427416

[35] Jack CR Jr, Andrews JS, Beach TG, et al Revised criteria for diagnosis and staging of Alzheimer’s disease: Alzheimer’s Association Workgroup. Alzheimers Dement. 2024;20:5143–5169. 38934362 10.1002/alz.13859 PMC11350039

[36] Zetterberg H, Bendlin BB. Biomarkers for Alzheimer’s disease—preparing for a new era of disease-modifying therapies. Mol Psychiatry. 2021;26:296–308. 32251378 10.1038/s41380-020-0721-9 PMC8172244

[37] Montplaisir J, Gagnon JF, Fantini ML, et al Polysomnographic diagnosis of idiopathic REM sleep behavior disorder. Mov Disord. 2010;25:2044–2051. 20818653 10.1002/mds.23257

[38] American Academy of Medicine . International classification of sleep disorders—3. American Academy of Sleep Medicine; 2014.

[39] Postuma RB, Berg D, Stern M, et al MDS clinical diagnostic criteria for Parkinson’s disease. Mov Disord. 2015;30:1591–1601. 26474316 10.1002/mds.26424

[40] Wenning GK, Stankovic I, Vignatelli L, et al The Movement Disorder Society criteria for the diagnosis of multiple system atrophy. Mov Disord. 2022;37:1131–1148. 35445419 10.1002/mds.29005 PMC9321158

[41] McKeith IG, Boeve BF, Dickson DW, et al Diagnosis and management of dementia with Lewy bodies: Fourth consensus report of the DLB consortium. Neurology. 2017;89:88–100. 28592453 10.1212/WNL.0000000000004058 PMC5496518

[42] Postuma RB, Gagnon J-F, Bertrand J-A, Marchand G, Montplaisir D, Montplaisir JY. Parkinson risk in idiopathic REM sleep behavior disorder: Preparing for neuroprotective trials. Neurology. 2015;84:1104–1113. 25681454 10.1212/WNL.0000000000001364 PMC4371408

[43] Nasreddine ZS, Phillips NA, Bedirian V, et al The Montreal cognitive assessment, MoCA: A brief screening tool for mild cognitive impairment. J Am Geriatr Soc. 2005;53:695–699. 15817019 10.1111/j.1532-5415.2005.53221.x

[44] APA PsycTests . Army individual test battery: Manual of directions and scoring. War Department, Adjutant General’s Office; 1944.

[45] Wechsler D . WAIS-III: Administration and scoring manual: Wechsler adult intelligence scale. Psychological Corporation; 1997.

[46] Delis DC, Kaplan EF, Kramer JH. Delis-Kaplan executive function system: Technical manual. The Psychological Corporation; 2001.

[47] Benton A, Sivan A, Hamsher K, Varney N, Spreen O. Contributions to neuropsychological assessment. Oxford University Press; 1983.

[48] Lucas JA, Ivnik RJ, Smith GE, et al Mayo’s older Americans normative studies: Category fluency norms. J Clin Exp Neuropsychol. 1998;20:194–200. 9777473 10.1076/jcen.20.2.194.1173

[49] Rey A . L’examen clinique en psychologie. Presses Universitaires de France; 1964.

[50] Rey A . L’examen psychologique dans les cas d’encephalopathie traumatique. Arch Psychol. 1941;28:286–340.

[51] Farnsworth D . The Farnsworth–Munsell 100 hue test and dichotomous tests for colour vision. J Opt Soc Am. 1943;33:568–578.

[52] Gagnon JF, Vendette M, Postuma RB, et al Mild cognitive impairment in rapid eye movement sleep behavior disorder and Parkinson’s disease. Ann Neurol. 2009;66:39–47. 19670440 10.1002/ana.21680

[53] Purcell S, Neale B, Todd-Brown K, et al PLINK: A tool set for whole-genome association and population-based linkage analyses. Am J Hum Genet. 2007;81:559–575. 17701901 10.1086/519795 PMC1950838

[54] Taliun D, Harris DN, Kessler MD, et al Sequencing of 53,831 diverse genomes from the NHLBI TOPMed program. Nature. 2021;590:290–299. 33568819 10.1038/s41586-021-03205-y PMC7875770

[55] Das S, Forer L, Schonherr S, et al Next-generation genotype imputation service and methods. Nat Genet. 2016;48:1284–1287. 27571263 10.1038/ng.3656 PMC5157836

[56] Ashton NJ, Brum WS, Di Molfetta G, et al Diagnostic accuracy of a plasma phosphorylated tau 217 immunoassay for Alzheimer disease pathology. JAMA Neurol. 2024;81:255–263. 38252443 10.1001/jamaneurol.2023.5319 PMC10804282

[57] Altomare D, Stampacchia S, Ribaldi F, et al Plasma biomarkers for Alzheimer’s disease: A field-test in a memory clinic. J Neurol Neurosurg Psychiatry. 2023;94:420–427. 37012066 10.1136/jnnp-2022-330619 PMC10314086

[58] Donaghy PC, Firbank M, Petrides G, et al The relationship between plasma biomarkers and amyloid PET in dementia with Lewy bodies. Parkinsonism Relat Disord. 2022;101:111–116. 35872565 10.1016/j.parkreldis.2022.07.008

[59] Genier Marchand D, Postuma RB, Escudier F, et al How does dementia with Lewy bodies start? Prodromal cognitive changes in REM sleep behavior disorder. Ann Neurol. 2018;83:1016–1026. 29665124 10.1002/ana.25239

[60] Jack CR Jr, Bennett DA, Blennow K, et al A/T/N: An unbiased descriptive classification scheme for Alzheimer disease biomarkers. Neurology. 2016;87:539–547. 27371494 10.1212/WNL.0000000000002923 PMC4970664

[61] Dickson DW, Heckman MG, Murray ME, et al *APOE* ε4 is associated with severity of Lewy body pathology independent of Alzheimer pathology. Neurology. 2018;91:e1182–e1195. 30143564 10.1212/WNL.0000000000006212 PMC6161556

[62] Schaffert J, LoBue C, White CL III, et al Risk factors for earlier dementia onset in autopsy-confirmed Alzheimer’s disease, mixed Alzheimer’s with Lewy bodies, and pure Lewy body disease. Alzheimers Dement. 2020;16:524–530. 32043803 10.1002/alz.12049 PMC7067630

[63] Bousiges O, Cretin B, Muller C, et al Involvement of *ApoE4* in dementia with Lewy bodies in the prodromal and demented stages: Evaluation of the Strasbourg cohort. Geroscience. 2024;46:1527–1542. 37653269 10.1007/s11357-023-00883-6 PMC10828291

[64] Boeve BF, Silber MH, Ferman TJ, et al Clinicopathologic correlations in 172 cases of rapid eye movement sleep behavior disorder with or without a coexisting neurologic disorder. Sleep Med. 2013;14:754–762. 23474058 10.1016/j.sleep.2012.10.015 PMC3745815

[65] Krohn L, Wu RYJ, Heilbron K, et al Fine-mapping of *SNCA* in rapid eye movement sleep behavior disorder and overt synucleinopathies. Ann Neurol. 2020;87:584–598. 31976583 10.1002/ana.25687 PMC8025046

[66] Van der Perren A, Gelders G, Fenyi A, et al The structural differences between patient-derived α-synuclein strains dictate characteristics of Parkinson’s disease, multiple system atrophy and dementia with Lewy bodies. Acta Neuropathol. 2020;139:977–1000. 32356200 10.1007/s00401-020-02157-3 PMC7244622

