# Supplementary material for: Plasma pTau181 and amyloid markers predict conversion to dementia in idiopathic REM sleep behaviour disorder
Source: Brain. 2025 Jan 6;148(6):2049–59. doi: 10.1093/brain/awaf003 (PMC12129744; doi:10.1093/brain/awaf003)
Supplement: awaf003_Supplementary_Data [file awaf003_supplementary_data.pdf]

Supplementary materials

Supplementary Tables

Supplementary Table 1. Baseline characteristics of the participants who completed follow-up vs. those who were lost to follow-up.

	Participants completed the study at censoring date or until phenoconversion	Participants lost to follow-up after blood sampling
**Number**	142	6
**Age, years**	67.6 ± 8.1	58.7 ± 5.2
**Sex, M/F**	106 / 33	4 / 2
**Education**	14.3 ± 3.6	16.6 ± 2.6
**MoCA**	26.3 ± 2.7	28.0 ± 1.1
**MDS-UPDRS III**	5.5 ± 4.7	3.5 ± 3.0

Supplementary Table 2. Cognitive features at moment of plasma sampling

	iRBD non-phenoconvertors	iRBD phenoconvertors	*p*-value[b]	DLB-convertors	PD-convertors	*p*-value[c]
**MoCA[a]**	27 [3]	26.5 [4]	0.52	25 [5]	28 [1]	0.02 *
**TMTA[a]**	0.203 [1.350]	-0.227 [1.147]	0.019 *	-0.383 [1.300]	0.126 [1.109]	0.08
**TMTB[a]**	-0.052 [1.872]	-0.960 [3.630]	0.028 *	-1.876 [2.983]	-0.030 [2.070]	0.005 *
**TMT B-A[a]**	0.150 [1.349]	-0.373 [2.134]	0.08	-1.627 [2.548]	0.412 [0.874]	0.002 *
**Stroop interference[a]**	0.330 [0.660]	0.330 [0.990]	0.62	0.330 [1.320]	0.330 [0.660]	0.54
**Stroop flexibility[a]**	0.330 [0.990]	0.000 [0.660]	0.07	0.000 [0.990]	0.000 [0.330]	0.19
**Digit Span forward**	0.029 ± 0.839	0.010 ± 0.911	0.92	0.004 ± 0.964	-0.006 ± 0.904	0.99
**Digit Span backward**	0.127 ± 0.962	0.279 ± 0.913	0.42	0.231 ± 0.948	0.246 ± 0.860	0.48
**RAVLT-total**	0.064 ± 1.339	-0.679 ± 1.451	0.013 *	-1.160 ± 1.318	-0.130 ± 1.453	0.005 *
**RAVLT-B**	-0.421 ± 1.114	-0.597 ± 0.964	0.39	-0.735 ± 0.865	-0.505 ± 1.078	0.49
**RAVLT-IR**	-0.171 ± 0.980	-0.856 ± 1.190	0.005 *	-1.308 ± 1.175	-0.342 ± 0.967	0.0002 *
**RAVLT-DR**	0.012 ± 1.097	-0.578 ± 1.388	0.033 *	-1.097 ± 1.318	0.092 ± 1.260	0.002 *
**RAVLT-recognition[a]**	0.385 [1.518]	-0.292 [2.000]	0.014 *	-0.867 [2.064]	0.385 [2.319]	0.034 *
**RCFT[a]**	0.276 [1.086]	0.089 [2.020]	0.31	-0.614 [2.896]	0.525 [1.169]	0.07
**Phonemic verbal fluency**	-0.277 ± 0.871	-0.227 ± 1.152	0.82	-0.392 ± 0.961	-0.186 ± 1.272	0.088
**Semantic verbal fluency**	-0.886 ± 0.608	-1.040 ± 0.928	0.39	-1.408 ± 0.852	-0.614 ± 0.869	0.006 *
**FM-100[a]**	117 [91]	160 [105]	0.005 *	150 [144]	160 [37]	0.03 *

Data shown as mean ± SD or median [IQR].[a] All cognitive test scores – with exception of MoCA and FM-100 - are presented as z-scores, adjusted for age, sex, and education depending on the normative data.

[b]*P*-values show comparison between iRBD participants without and with phenoconversion during follow-up with Student's *t*-test, or Mann-Withney U test.[a] Threshold for significance $\alpha < 0.05$. Significant p-values are marked with *.

[c]*P*-values show comparison between iRBD participants without phenoconversion, DLB- and PD-convertors, with ANOVA or Kruskal-Wallis test.[a] Threshold for significance $\alpha < 0.05$. Significant p-values are marked with *.

iRBD, idiopathic/isolated REM sleep behavior disorder; DLB, dementia with Lewy bodies; PD, Parkinson's disease; MoCA, Montreal Cognitive Assessment; TMT, Trail Making Test; RAVLT, Rey Auditory Verbal Learning Test; RAVLT-total, trials 1 to 5; RAVLT-B, list B; RAVLT-IR, immediate recall; RAVLT-DR, delayed recall; RAVLT-RC, recognition; RCFT, Rey-Osterrieth Complex Figure Test; FM-100, Farnsworth-Munsell 100-hue test.

Supplementary Table 3. Plasma levels of AD-related markers stratified by MoCA score.

Plasma biomarker	Aβ42/40 ratio	*p*-value *t*-test	pTau181, pg/ml	*p*-value *t*-test
**Non-phenoconvertors**				
• Normal MoCA score (n=75)	0.114 ± 0.012	(reference)	0.741 ± 0.215	(reference)
• Abnormal MoCA score (n=35)	0.112 ± 0.012	(reference)	0.876 ± 0.336	(reference)
**Phenoconvertors**				
• Normal MoCA score (n=18)	0.100 ± 0.009	< 0.001 *	0.920 ± 0.321	0.04 *
• Abnormal MoCA score (n=13)	0.106 ± 0.010	0.08	1.130 ± 0.369	0.04 *
**DLB-convertors**				
• Normal MoCA score (n=7)	0.095 ± 0.009	< 0.001 *	1.130 ± 0.356	0.03 *
• Abnormal MoCA score (n=11)	0.105 ± 0.010	0.04 *	1.160 ± 0.323	0.02 *
**PD-convertors**				
• Normal MoCA score (n=11)	0.104 ± 0.008	0.001 *	0.789 ± 0.226	0.52
• Abnormal MoCA score (n=2)	0.119 ± 0.000	NA (n too low)	0.966 ± 0.727	NA (n too low)

Plasma levels of primary plasma markers Aβ42/40 ratio and pTau181. Unpaired Student's t-test was used for comparisons of the phenoconvertors, DLB- and PD-convertors with the non-phenoconvertors (reference group) of the respectively MoCA status group (normal MoCA [n = 93] vs. abnormal MoCA scores [n = 47]) at time of blood sampling.

Supplementary Table 4. Outcomes of ROC analysis for Aβ42/40 ratio and pTau181 for DLB-convertors compared to non-phenoconvertors without MCI

**Aβ42/40 ratio**		
AUC	0.835	95%CI [0.736, 0.933]
**Optimal threshold:** Ab42/40 ratio = 0.112		
Sensitivity	88.9 %	95%CI [65.3; 98.6%]
Specificity	63.4 %	95%CI [52.1; 73.8%]
PPV	34.8 %	95%CI [27.8; 42.6%]
NPV	96.3 %	95%CI [87.5; 99.0%]
**pTau181**		
AUC	0.848	95%CI [0.731, 0.964]
**Optimal threshold:** pTau181 = 0.802 pg/ml		
Sensitivity	88.9 %	95%CI [65.3; 98.6%]
Specificity	73.2%	95%CI [62.2; 82.4%]
PPV	42.1%	95%CI [32.9; 51.9%]
NPV	96.8%	95%CI [89.0; 99.1%]

AUC, area under the curve; PPV, positive predictive value; NPV, negative predictive value; ROC, ; DLB, dementia with Lewy bodies; MCI, mild cognitive impairment.

Supplementary Table 5. Time-to-event analysis using quantiles

<table>
<tr><th>A.</th><th colspan="3">pTau181</th></tr>
<tr><td></td><td>Group size, cut-offs and no. events</td><td>Hazard ratio</td><td>Kaplan-Meier Time-to-event plot</td></tr>
<tr><td>2-quantiles (median split)</td><td>n = 50

Q1
pTau181
< 0.718
<u>events</u>: 2

Q2
pTau181
> 0.718
<u>events</u>: 16</td><td>HR = 8.89

95%CI [2.03; 38.75]</td><td>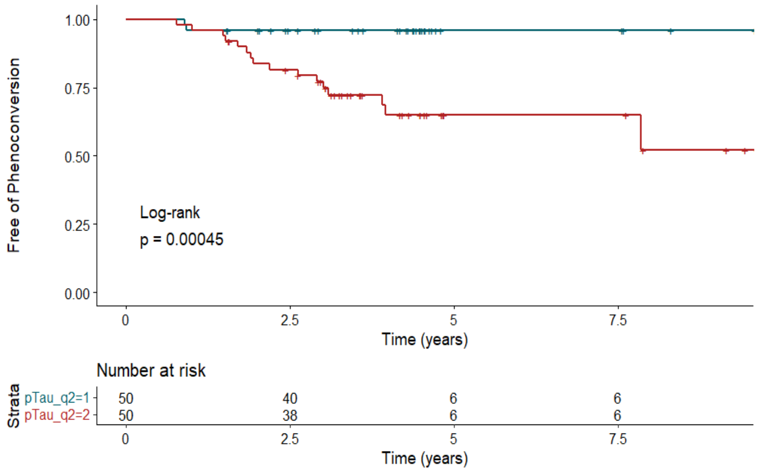
</td></tr>
<tr><td>3-quantiles (tertiles)</td><td>n = 33 or 34

Q1
pTau181
< 0.657
<u>events</u>: 1

Q2
pTau181
0.658-0.900
<u>events</u>: 2

Q3
pTau181
> 0.900
<u>events</u>: 15</td><td>HR = 6.25

95%CI [2.36; 16.60]</td><td>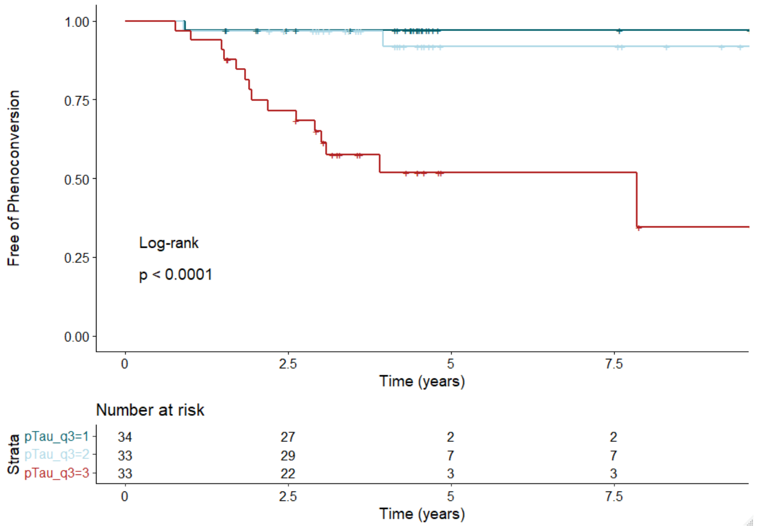
</td></tr>
<tr><td>4-quantiles (quartiles)</td><td>n = 25

Q1
pTau181
< 0.612
<u>events</u>: 1

Q2
pTau181
0.612-0.718
<u>events</u>: 1

Q3
pTau181
0.718-0.992
<u>events</u>: 3

Q4
pTau181
> 0.992
<u>events</u>: 13</td><td>HR = 3.74

95%CI [1.90; 7.33]</td><td>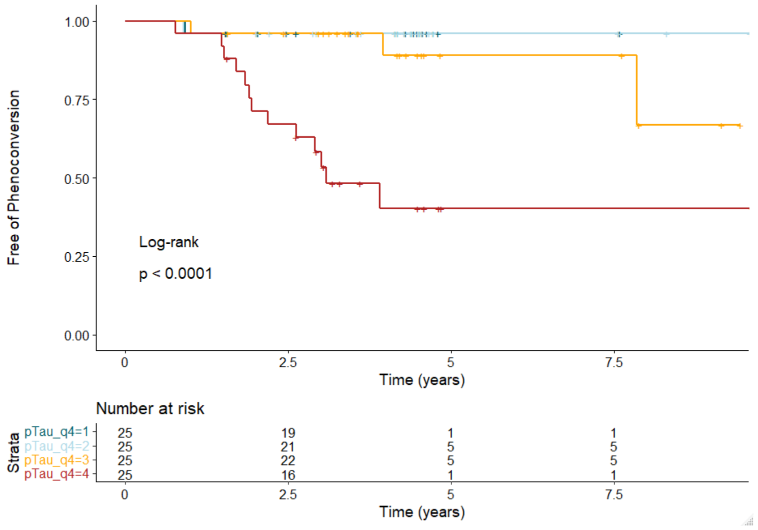
</td></tr>
</table>

**B.**			**Aβ42/40**
	**Group size, cut-offs and no. events**	**Hazard ratio**	**Kaplan-Meier Time-to-event plot**
**2-quantiles (median split)**	n = 50<br><br>**Q1**<br>Aβ42/40<br>< 0.113<br>events: 16<br><br>**Q2**<br>Aβ42/40<br>> 0.113<br>events: 2	HR = 8.48<br><br>95%CI[1.95; 36.95]	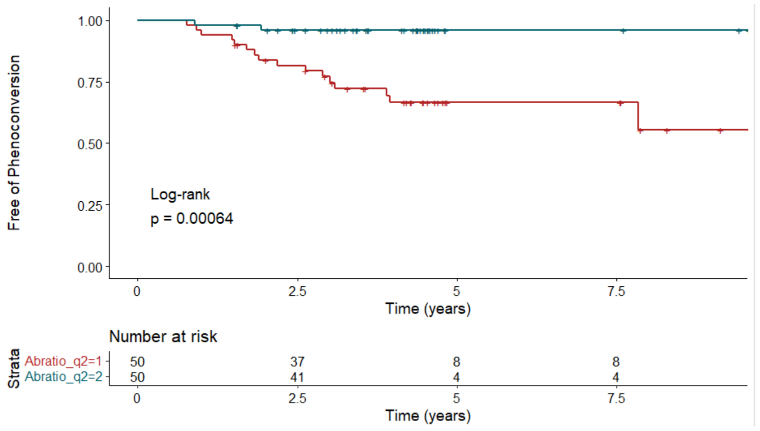
**3-quantiles (tertiles)**	n = 33 or 34<br><br>**Q1**<br>Aβ42/40<br>< 0.108<br>events: 13<br><br>**Q2**<br>Aβ42/40<br>0.108-0.119<br>events: 5<br><br>**Q3**<br>Aβ42/40<br>> 0.119<br>events: 0	HR = 4.59<br><br>95%CI [1.94; 10.91]	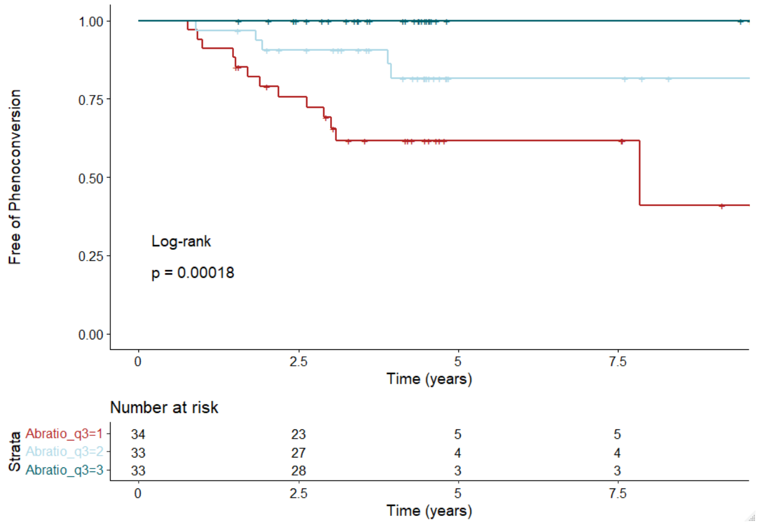
**4-quantiles (quartiles)**	n = 25<br><br>**Q1**<br>Aβ42/40<br>< 0.105<br>events: 13<br><br>**Q2**<br>Aβ42/40<br>0.105- 0.113<br>events: 3<br><br>**Q3**<br>Aβ42/40<br>0.113-0.121<br>events: 2<br><br>**Q4**<br>Aβ42/40<br>> 0.121<br>events: 0	HR = 3.94<br><br>95%CI [1.95; 7.98]	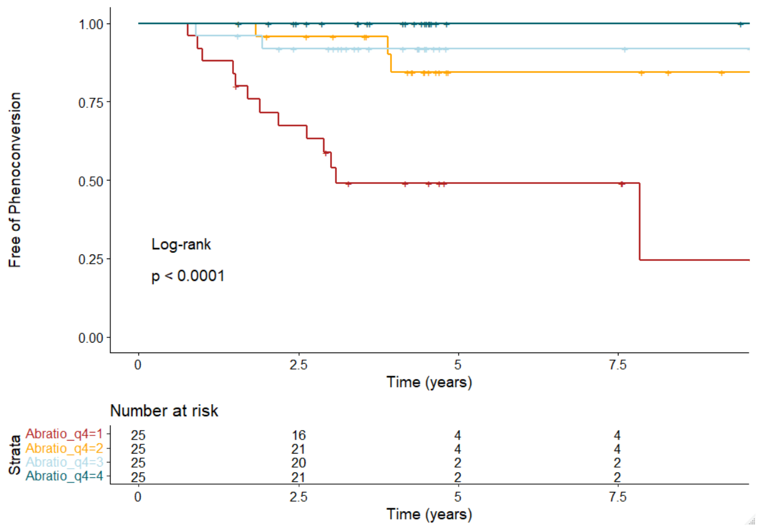

Supplementary Table 6. Outcomes of regression analysis for plasma Aβ42/40 or pTau181 for cognitive testing

Cognitive tests	Intercept estimate	β-coefficient	SE	*p*-value
**Aβ42/40 ratio**				
MoCA	0.152	-0.0001	0.0004	0.71
Attention				
Digit span forward	0.142	0.0003	0.0012	0.80
Digit span backward	0.142	-0.0002	0.0011	0.84
Stroop interference	0.142	0.0038	0.0011	< 0.001*
TMT A	0.140	0.0012	0.0009	0.22
Verbal learning				
RAVLT-total	0.142	0.0002	0.0007	0.83
RAVLT-B	0.142	0.0015	0.0009	0.11
RAVLT-IR	0.142	0.0012	0.0009	0.22
RAVLT-DR	0.142	0.0011	0.0008	0.18
RAVLT-RC	0.141	0.0011	0.0008	0.17
Executive function				
TMT B	0.141	0.0004	0.0006	0.57
TMT part B minus A	0.142	0.0001	0.0007	0.86
Stroop flexibility	0.143	-0.0005	0.0013	0.69
Phonemic verbal fluency	0.143	-0.0002	0.0011	0.83
Semantic verbal fluency	0.143	0.0005	0.0015	0.73
Visuospatial, RCFT	0.140	0.0012	0.0009	0.19
Color vision, FM-100	0.143	-0.0000	0.00001	0.69
**pTau181**				
MoCA	0.596	-0.025	0.009	0.005*
Attention				
Digit span forward	-0.159	-0.037	0.028	0.19
Digit span backward	-0.079	-0.050	0.025	0.048*
Stroop interference	-0.142	-0.016	0.027	0.56
TMT A	-0.112	-0.032	0.022	0.14
Verbal learning				
RAVLT-total	-0.190	-0.054	0.016	0.001*
RAVLT-B	-0.164	-0.048	0.022	0.03*
RAVLT-IR	-0.137	-0.068	0.022	0.002*
RAVLT-DR	-0.178	-0.061	0.019	0.002*
RAVLT-RC	-0.136	-0.048	0.018	0.009*
Executive function				
TMT B	-0.094	-0.028	0.015	0.06
TMT part B minus A	-0.102	-0.037	0.016	0.02*
Stroop flexibility	-0.018	-0.049	0.030	0.10
Phonemic verbal fluency	-0.174	-0.050	0.025	0.049*
Semantic verbal fluency	-0.186	-0.082	0.035	0.02*
Visuospatial, RCFT	-0.052	-0.062	0.021	0.005*
Color vision, FM-100	-0.073	0.0007	0.0003	0.04*

Linear regression models adjusted for age and sex. Threshold for significance $\alpha < 0.05$. Significant p-values are marked with *.
MoCA, Montreal Cognitive Assessment; TMT, Trail Making Test; RAVLT, Rey Auditory Verbal Learning Test; RAVLT-total, trials 1 to 5; RAVLT-B, list B; RAVLT-IR, immediate recall; RAVLT-DR, delayed recall; RAVLT-RC, recognition; RCFT, Rey-Osterrieth Complex Figure Test; FM-100, Farnsworth-Munsell 100-hue test.

Supplementary Table 7. Plasma AD biomarkers stratified by *ApoE* status

**Biomarker**	**No *ApoEε4* allele n=85**	***ApoEε4* carrier n=21**	***p*-value**	**Cohen-d[a]**
**Aβ40** (pg/ml)	241.0 ± 74.9	205.0 ± 53.4	0.10	0.50
**Aβ42** (pg/ml)	26.89 ± 8.11	21.37 ± 5.98	0.01*	0.71
**Aβ42/40 ratio**	0.112 ± 0.012	0.104 ± 0.011	0.0007*	0.70
**pTau181** (pg/ml)	0.836 ± 0.295	0.848 ± 0.314	0.35	0.04

Mean ± SD group value for the different plasma biomarkers stratified by *ApoEε4* carrier status. ANOVA with correction for age and sex was used for group comparisons. Threshold for significance $\alpha < 0.05$. Significant p-values are marked with *.
[a]Cohen d effect size unadjusted for age and sex
AD, Alzheimer's disease; iRBD, idiopathic/isolated REM sleep behavior disorder; SD, standard deviation; pTau181, tau phosphorylated at threonine 181.

Supplementary Figures

Supplementary Fig. 1. Flow diagram.

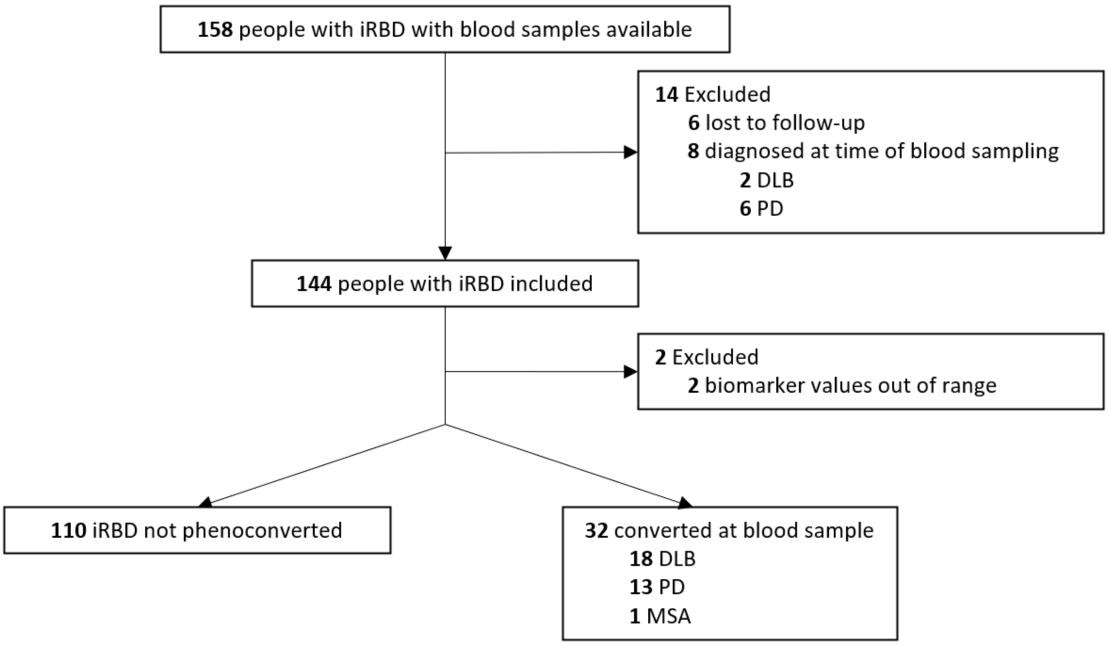

Supplementary Fig. 2. Biomarker levels comparing different early synucleinopathies

Boxplots and individual levels for different plasma biomarkers in iRBD subgroups: non-phenoconvertors, DLB-convertors, PD-convertors without MCI and PD-convertors with MCI. * Significantly different from non-phenoconvertors.

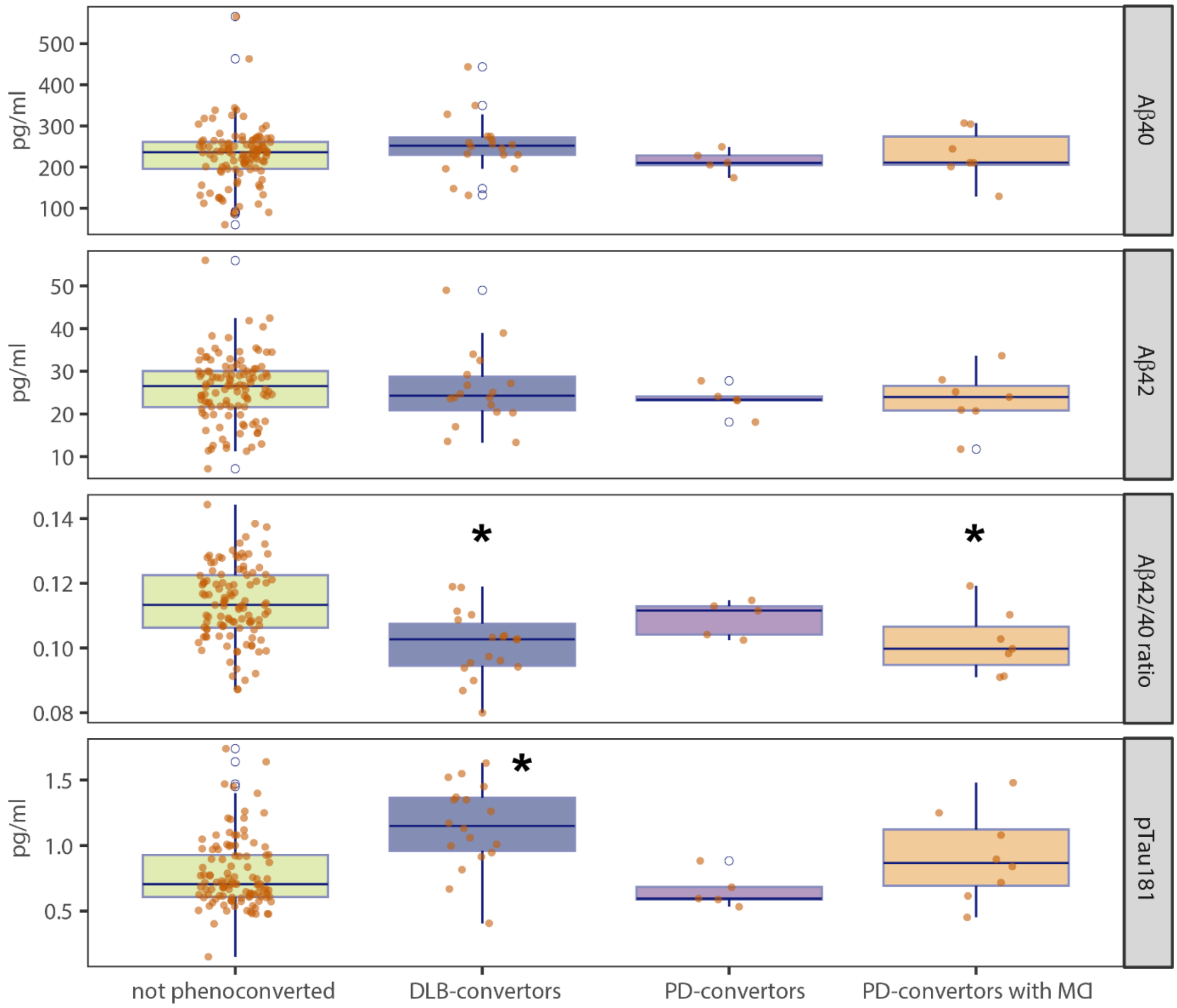

Supplementary Fig. 3. Correlations between different plasma biomarkers in iRBD

Scatterplot with linear regression showing correlation between plasma pTau181 and Aβ42/40 ratio in the total iRBD cohort.

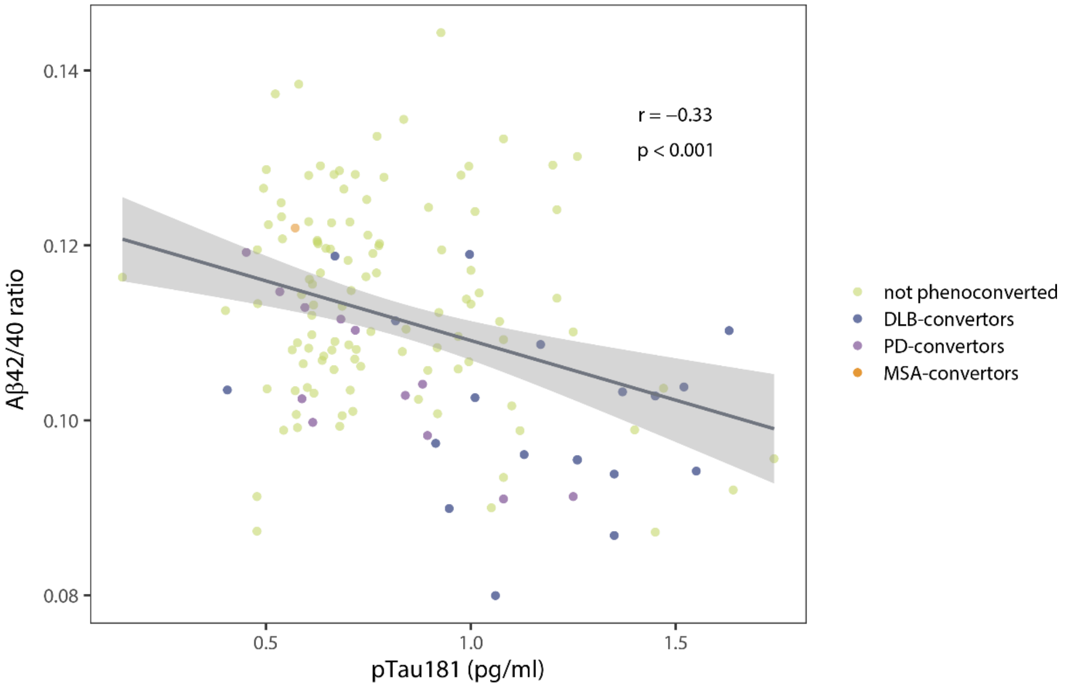

Supplementary Fig. 4. Biomarker levels stratified by MoCA status

(A) MoCA scores in the different iRBD subgroups and **(B, C)** plasma Aβ42/40 ratio and pTau181 levels stratified by MoCA normal vs. abnormal scores, showing the distribution of the different iRBD subgroups (non-phenoconvertors, DLB- and PD-convertors).

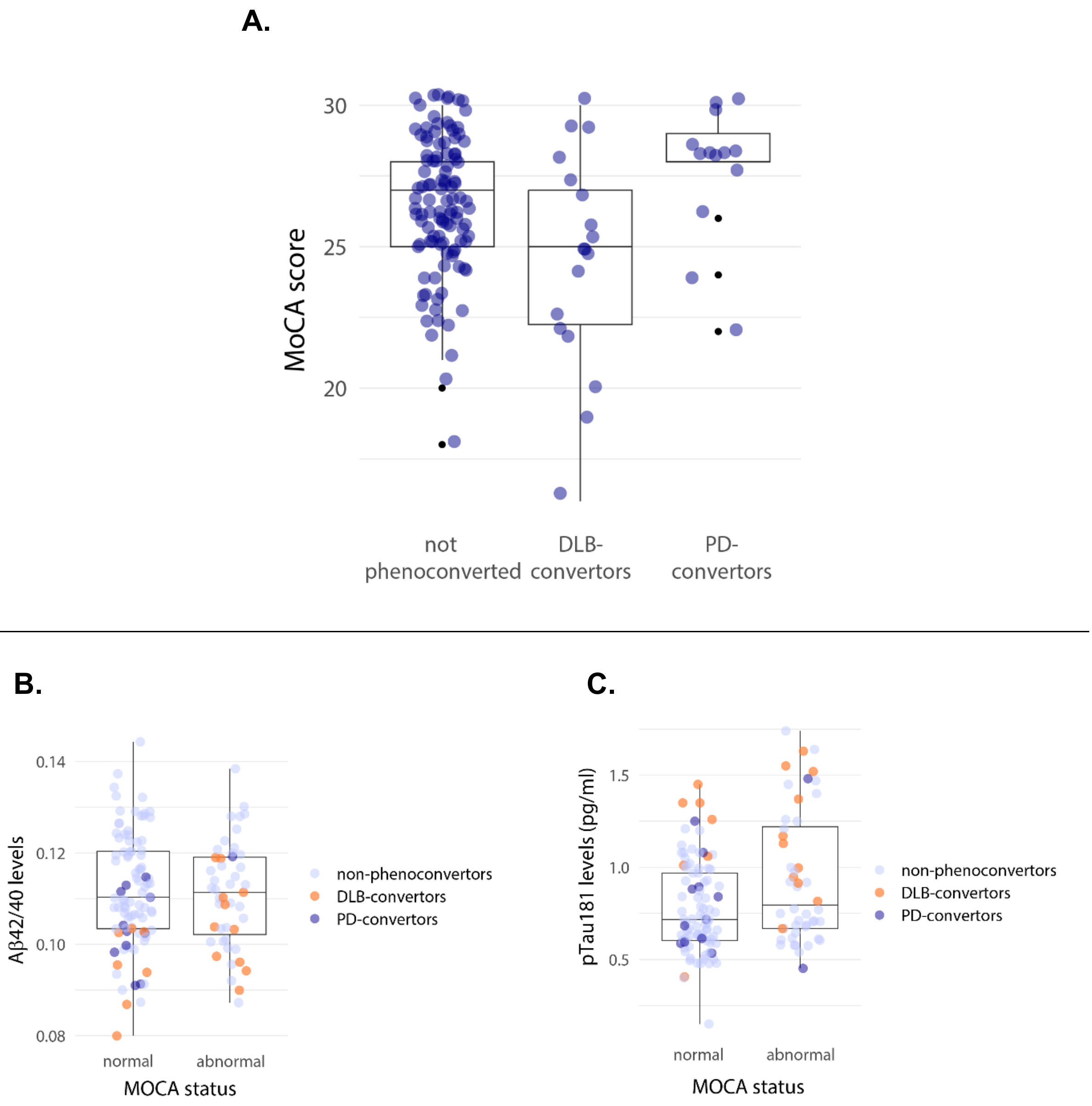

Supplementary Fig. 5. ROC curves analysis of plasma AD-related biomarkers

Receiver operating characteristic (ROC) curves analysis for plasma Aβ42/40 ratio and pTau181 in DLB-convertors, with a reference group of non-phenoconvertors without mild cognitive impairment up to the most recent follow-up.

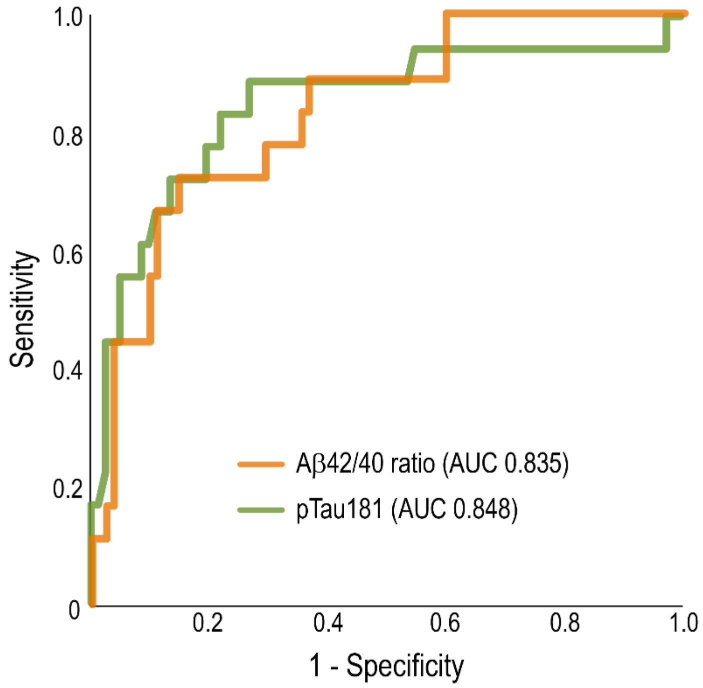

Supplementary Fig. 6. Biomarker levels stratified by *ApoE* status

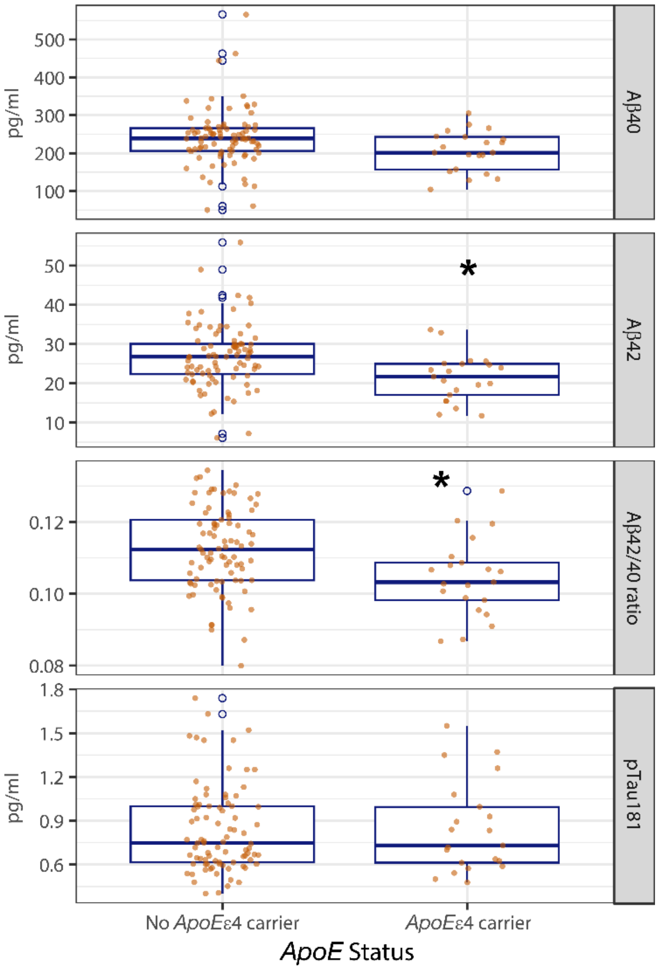

Boxplots and individual levels for different plasma AD-related biomarkers in *ApoEε4* non-carriers and *ApoEε4* carriers. * Significantly different from *ApoEε4* non-carriers.
